# Assessment of fecal bacterial viability and diversity in fresh and frozen fecal microbiota transplant (FMT) product in horses

**DOI:** 10.1186/s12917-024-04166-w

**Published:** 2024-07-10

**Authors:** Alicia E. Long, Dipti Pitta, Meagan Hennessy, Nagaraju Indugu, Bonnie Vecchiarelli, Daniela Luethy, Helen Aceto, Samuel Hurcombe

**Affiliations:** 1 Department of Clinical Studies, New Bolton Center, University of Pennsylvania, Kennett Square, PA USA; 2 Veterinary Innovative Partners, New York, NY USA

**Keywords:** Microbiota, Microbiome, Fecal microbiota transplant, Horse, Fecal storage, Buffer

## Abstract

**Background:**

Currently, lack of standardization for fecal microbiota transplantation (FMT) in equine practice has resulted in highly variable techniques, and there is no data on the bacterial metabolic activity or viability of the administered product. The objectives of this study were to compare the total and potentially metabolically active bacterial populations in equine FMT, and assess the effect of different frozen storage times, buffers, and temperatures on an equine FMT product. Fresh feces collected from three healthy adult horses was subjected to different storage methods. This included different preservation solutions (saline plus glycerol or saline only), temperature (-20 °C or -80 °C), and time (fresh, 30, 60, or 90 days). Samples underwent DNA extraction to assess total bacterial populations (both live and dead combined) and RNA extraction followed by reverse transcription to cDNA as a proxy to assess viable bacteria, then 16s rRNA gene amplicon sequencing using the V1-V2 region.

**Results:**

The largest difference in population indices and taxonomic composition at the genus level was seen when evaluating the results of DNA-based (total) and cDNA-based (potentially metabolically active) extraction method. At the community level, alpha diversity (observed species, Shannon diversity) was significantly decreased in frozen samples for DNA-based analysis (*P* < 0.05), with less difference seen for cDNA-based sequencing. Using DNA-based analysis, length of storage had a significant impact (*P* < 0.05) on the bacterial community profiles. For potentially metabolically active populations, storage overall had less of an effect on the bacterial community composition, with a significant effect of buffer (*P* < 0.05). Individual horse had the most significant effect within both DNA and cDNA bacterial communities.

**Conclusions:**

Frozen storage of equine FMT material can preserve potentially metabolically active bacteria of the equine fecal microbiome, with saline plus glycerol preservation more effective than saline alone. Larger studies are needed to determine if these findings apply to other individual horses. The ability to freeze FMT material for use in equine patients could allow for easier clinical use of fecal transplant in horses with disturbances in their intestinal microbiome.

**Supplementary Information:**

The online version contains supplementary material available at 10.1186/s12917-024-04166-w.

## Background

Fecal microbiota transplantation (FMT) is a practice currently used in human medicine as part of the management of *Clostridiodes difficile* infection (CDI), inflammatory bowel disease (IBD), and other problems association with disruption of the healthy intestinal microbiome [1–5]. FMT involves infusion of a liquid suspension of donor stool with the goal of transferring the donor’s healthy gut microbiota to the patient to allow for a healthy colonic microbiota and colonization resistance against pathogens. Multiple studies have evaluated the use of FMT for patients with recurrent CDI and have demonstrated low rates of recurrence and improved cure rates compared to conventional antibiotic treatment [1, 6–10]. The use of FMT as a part of multimodal therapy for IBD in humans has been investigated in several studies, with clinical remission rates ranging from 22 to 60% in several meta-analyses [5]. In veterinary medicine, data regarding the use of FMT, particularly in horses, is currently limited [11].

The large intestine of the horse, a species classified as a hindgut fermenter, is a fermentation chamber colonized by microbes that help digest indigestible plant fiber. The horse’s diet comprises a significant portion of plant material to allow microbial fermentation in the large intestine and provide for most of the host’s energy requirements. In humans, a healthy intestinal microbiome is also critical for proper gastrointestinal health by modulating the immune response, enteric neurologic signaling, and endocrine function [12, 13]. It is likely the equine intestinal microbiome plays a similar role in overall intestinal health in addition to its fundamental role in digestion and metabolism. Previous literature, although limited, has demonstrated differences in the fecal microbiota between healthy and diseased horses, both in cases of gastrointestinal disease (e.g. colic, colitis) and non-gastrointestinal conditions (e.g. equine metabolic syndrome and asthma) [14–19]. Since knowledge of the role of the gut microbiome in intestinal related problems in the horse is just emerging, it is not yet clear the extent of microbial dysbiosis in horses with colic, or the impact of clinical interventions on reverting the dysbiosis to near normalcy.

In horses, only a small number of published studies exist regarding the use of FMT in normal or diseased horses, with small sample sizes and lack of appropriate controls making conclusions on efficacy difficult and giving variable results on clinical outcome following FMT [20–27]. For studies evaluating clinical efficacy of FMT in patients with gastrointestinal disease, the majority report improvement in clinical signs [20–24], while one reported variable response between individual animals [25]. However, in four studies in which the fecal microbiota was evaluated, there was no difference seen post FMT administration or when compared to controls [22–26], while two showed significant differences in bacterial communities after FMT with again variable response between individuals [21, 27]. Despite inherent limitations such as fewer studies and small sample size to test the efficacy of FMT in horses, research points to the large variation commonly noted in the microbiomes of individual horses [28]. This is not normally accounted for in equine microbial analysis and could certainly be a reason for variable efficacy of FMT. Thus, it becomes pertinent to evaluate the quality of FMT product even before its use in recipient horses. Donor availability for FMT can be difficult in equine medicine due to the lack of healthy donor or lack of easily accessible donor close enough to the patient to provide fresh feces. Therefore, storage of an FMT product becomes the most feasible and practical option. There is no available data in horses evaluating FMT material or the effects of preparation or storage of equine FMT.

In human medicine, there remain unanswered questions regarding best practices for preparation, handling, and administration of donor material. Initially, most studies evaluating efficacy of FMT in humans utilized fresh transplant material, which is collected, processed, and administered to the patient immediately [1, 8]. In contrast, more recent studies have assessed the utility of frozen fecal transplant material for FMT for ease of preparation and administration. Several controlled trials have demonstrated no difference in efficacy between fresh and frozen transplant material for treatment of CDI [4, 7, 9]. While such studies offer promise, evidence to demonstrate the viability of fecal microbiota in the stored FMT product has largely been unexplored in human subjects and has never been tested to date in equines. One study of human fecal transplantation to mice did not show any difference in bacterial viability (DNA analysis of PMA-treated samples) in vitro between various lengths of storage of frozen material or multiple freeze-thaw cycles but did demonstrate decreased colonization ability of the bacteria when frozen for one month prior to transplant [29] suggesting that length of time is an important factor while storing FMT product. Furthermore, the temperature at which the FMT product is also an an important factor. Storing FMT product at -20 °C for greater than a week significantly decreases the viability of culturable bacterial populations [30]. In yet another study using equine feces, it was demonstrated that while the overall bacterial composition and their viability showed no differences, minor populations were altered with storage conditions, albeit only in one horse [31]. Collectively, these data imply that standard operating procedures that account for inter-animal variations and the effect of storage on microbial communities and individual taxa within a community of different horses are much needed to improve the quality and efficacy of FMT product.

The viability of the organism within an FMT product is key to the success of the treatment, and therefore the evaluation of whether bacteria and other organisms survive any processing or storage is essential. Several methods are utilized to assess potential viability in the fecal microbiome, including culture-based and genome-based methods (such as PMA-treated DNA analysis or RNA analysis). There have been studies in foregut fermenters such as dairy cattle that demonstrated very distinct differences in total (DNA-based) and potentially metabolically active (RNA reversed transcribed to cDNA-based) communities and their individual taxa using 16 S rDNA-based sequencing commonly employed for bacterial diversity studies [32]. The use of RNA as a marker of metabolic activity potential of organisms is based off the knowledge that only functional cells produce RNA as part of normal cell function and reproduction [33, 34]. To the authors’ knowledge, no previous study has used RNA-based analysis to assess the potential viability of bacteria in equine feces.

The first objective of this study was to assess if diversity indices and taxonomic composition results would differ between extraction methods, specifically DNA-based (total community) and cDNA-based (potentially metabolically active community)16 S rDNA sequencing of equine feces. The second objective was to determine if different frozen storage time points, buffers such as saline and saline plus glycerol, and temperatures such as -20 °C and − 80 °C to store FMT product would affect the total (DNA-based) and potentially metabolically active (cDNA-based) populations in feces of different horses. We hypothesized there will be differences in total and potentially metabolically active populations in fresh FMT product which will likely be affected differently by different storage conditions such as buffer, temperature, and length of storage time.

## Results

### Sample population and sequencing information

The sequenced data yielded a total of 7,488,167 raw reads from 125 samples (DNA:47; cDNA:78). After initial quality control and denoising with dada2, a total of 4,930,027 (66%) sequence-reads were produced. Less than 100 reads per sample were observed in the blank samples (two DNA blanks and three PCR blanks) and in one experimental sample (Horse 1, DNA, saline plus glycerol, -80 °C, Day 60), and this experimental sample was removed from the analysis.

### Community comparison

#### Alpha diversity (within sample variation)

**Fig. 1:**
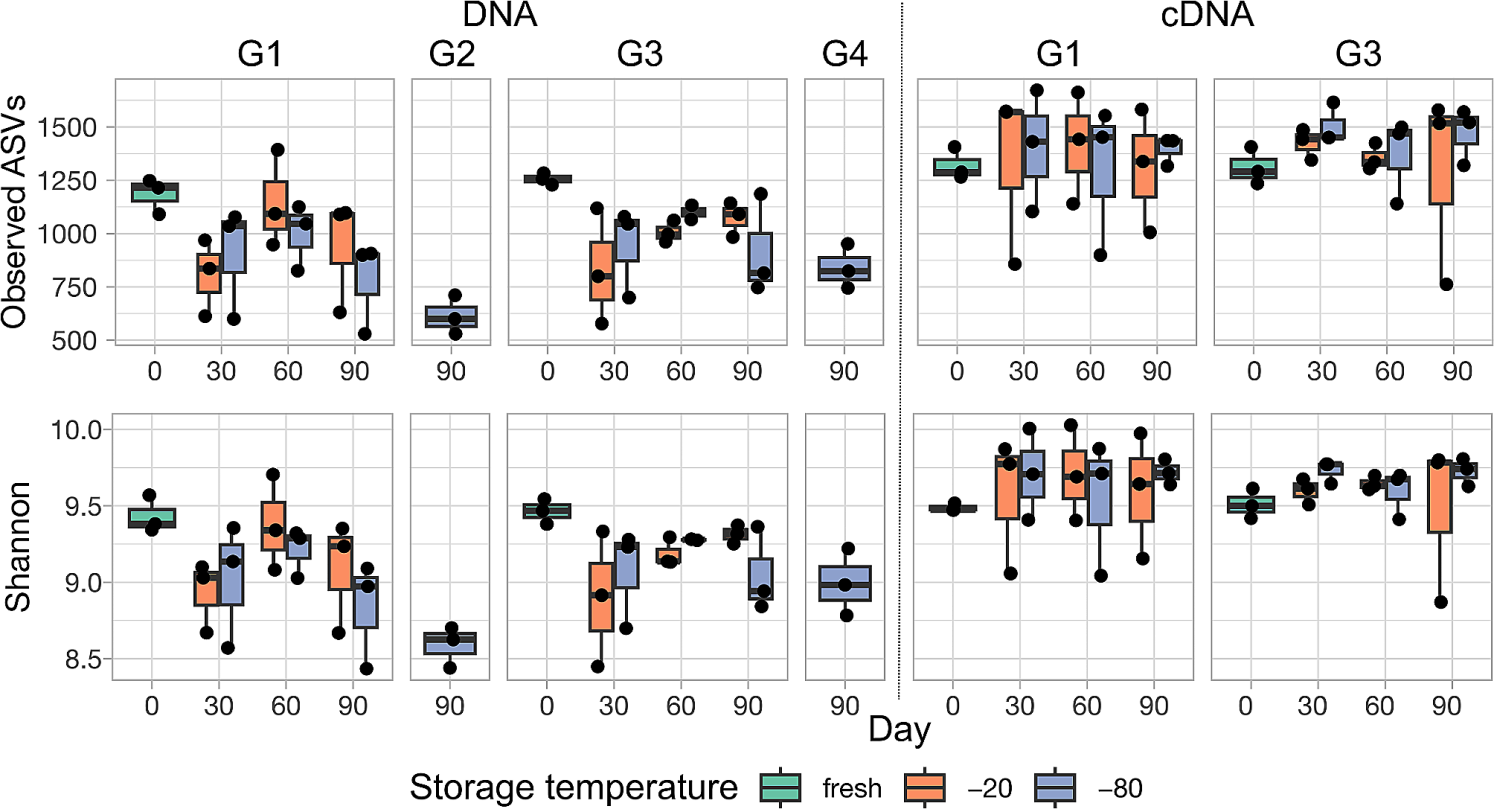
Measurement of alpha diversity indices (observed species, Shannon diversity) for DNA and cDNA-based analysis. G1 = small (300 µl or 1.5 ml) aliquots with saline only buffer; G2 = 1-liter aliquot with saline only buffer; G3 = small aliquots with saline plus glycerol buffer; G4 = 1-liter aliquot with saline plus glycerol buffer. X-axis shows the day of storage (Day 0 or fresh, 30, 60, or 90). The different colors represent the storage temperature (fresh, frozen at -20 °C, or frozen at -80 °C). Each box and whisker plot shows results for 3 samples (1 from each horse), as represented by the individual data points. Alpha diversity indices for cDNA-based analysis showed much less variation between day and temperature of storage (*P* > 0.05) than for DNA-based analysis (*P* < 0.001 for fixed effect of day for both − 20 °C and − 80 °C), and the frozen samples were overall more similar to fresh samples than for DNA-based analysis. There was less variation in alpha diversity between horse for cDNA-based analysis when using saline plus glycerol buffer as compared to saline only buffer. For the 1-liter aliquot size, less variation was seen when using saline plus glycerol buffer as opposed to saline only buffer (only DNA-based analysis performed)

Alpha diversity was represented using both species richness (observed amplicon sequence variants; ASV) and Shannon diversity indices and was compared within DNA and cDNA-based bacterial communities (Fig. 1). Within each extraction type, we performed analysis for temperature at -20 °C and − 80 °C separately as they were considered two different experiments (Additional Table 1). Within each temperature, we included buffer (saline and saline plus glycerol), day (day 0 or fresh, day 30, day 60, and day 90) and their interaction. As the variation among the three horses were noticeable, we included “horse” as the random variable in the model. Results showed no effect of buffer or day at either temperature in cDNA components for both Shannon diversity and species richness (Additional Table 1). However, for DNA-based analysis, day had a significant effect on both species richness and Shannon diversity at -80 °C (Additional Table 1).

#### Beta diversity (between sample variation)

**Fig. 2:**
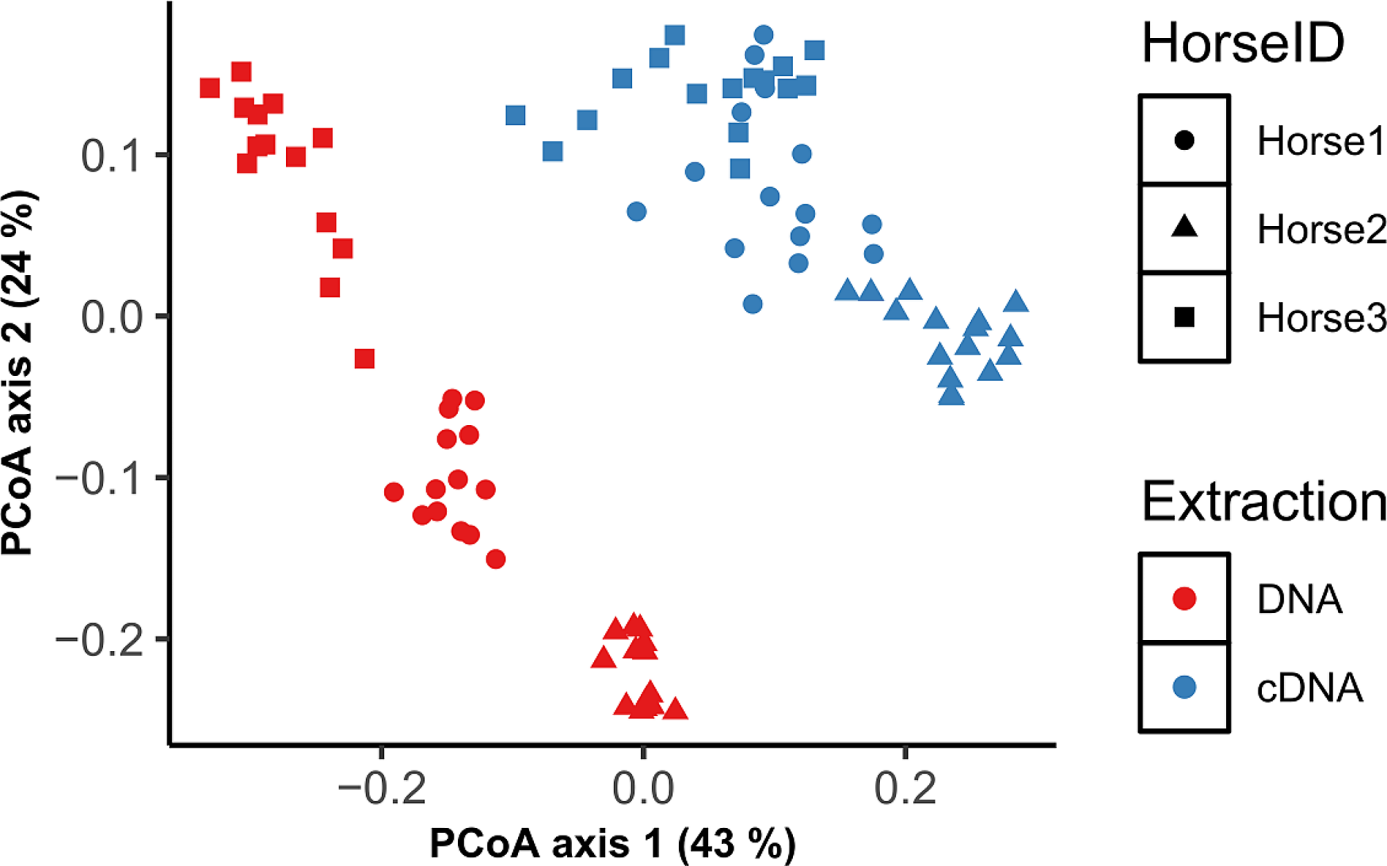
Measurement of bacterial community composition (beta diversity) for analysis type (DNA vs. cDNA) and horse. The principal coordinate analysis (PCoA) plot shows weighted UniFrac distances between samples, with samples that are more similar located closer to one another. Each data point represents an individual slurry sample. Differences in beta diversity were seen between extraction type and individual horses, as observed by the clustering of each horse within extraction type, with more variation within each horse for DNA-based analysis than cDNA-based analysis

**Fig. 3:**
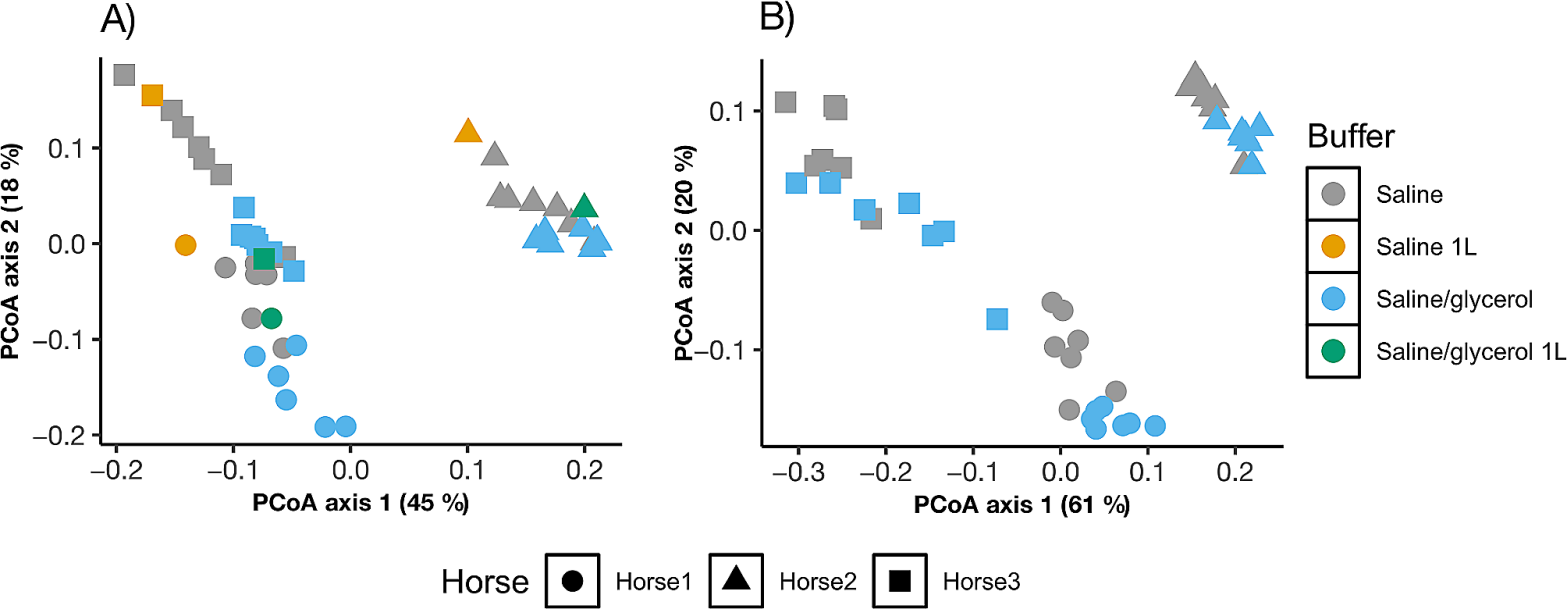
Measurement of bacterial community composition (beta diversity) for the storage variable buffer for DNA (A) and cDNA (B) extraction. The principal coordinate analysis (PCoA) plot shows weighted UniFrac distances between samples, with samples that are more similar located closer to one another. Each data point represents an individual slurry sample. For this study, there was an effect of individual horse for all variables within both DNA and cDNA analysis.Bacterial community composition was more similar within buffer type for both DNA and cDNA

The bacterial community composition of all fecal samples with phylogeny-based weighted (commonly present bacterial taxa) UniFrac distances using principal coordinates analysis (PCoA) is illustrated in Fig. 2 (unweighted in Additional Fig. 1). Like alpha diversity, we performed PERMANOVA analysis for DNA and cDNA-bacterial communities separately. Within each extraction type, analysis was separated for − 20 °C and − 80 °C temperatures. Results of PERMANOVA analysis for weighted and unweighted (uniquely present bacterial taxa) UniFrac distances are presented in Table 1 (main fixed effects) and Additional Table 6 (pairwise comparison). The bacterial communities clustered by extraction type, which is not surprising as differences between DNA and cDNA communities were expected in both fresh and stored samples. Within each extraction type, communities clustered by horse showing large differences (*P* < 0.001) in bacterial communities between horses. PCoA plots for weighted UniFrac distances by individual storage variables (buffer, temperature, length of storage) within each extraction method is shown (Fig. 3 and Additional Fig. 2, unweighted in Additional Fig. 3). Like alpha diversity, the effects of temperature, buffer and day were insignificant compared to differences between horses; however, when blocked by individual horse, then differences by buffer and day were noticed as described below.

**Table 1:** Effects of storage variables (buffer, day) and their interaction on beta diversity patterns within each frozen storage temperature and extraction type

	DNA				cDNA			
	-20		-80 °C		-20 °C		-80 °C	
	WU	UWU	WU	UWU	WU	UWU	WU	UWU
**Buffer**	0.002	0.092	0.003	0.009	0.001	0.002	0.008	0.296
**Day**	0.003	0.008	0.015	0.007	0.007	0.005	0.004	0.007
Buffer*Day	0.724	0.786	0.502	0.768	0.846	0.732	0.735	0.721

WU = weighted UniFrac distance, UWU = unweighted UniFrac distance,

Within DNA-based bacterial communities (Table 1, Additional Table 2), for weighted UniFrac distances, differences (*P* < 0.001) were observed by day and buffer at -20 °C, but only by day at -80 °C, although the R^2^ values (which indicate proportion of the variance) were small. Further, differences (*P* < 0.001) in bacterial communities were also noted between fresh and 1-liter bottle stored at -80 °C. For unweighted UniFrac distances, differences were observed only by day at -20 °C and by buffer (*P* < 0.003) and day (*P* < 0.01) at -80 °C. Comparing fresh to 1-liter bottle stored at -80 °C, bacterial communities differed (*P* < 0.001) by buffer, day, and their interaction. The R^2^ values for unweighted analysis were much lower compared to weighted analysis as the contribution of rare bacterial populations are much smaller compared to commonly present populations.

Within cDNA-based bacterial communities (Table 1, Additional Table 2), for both weighted and unweighted UniFrac distances, differences (*P* < 0.001) in bacterial communities were observed by day and buffer at -20 °C and at -80 °C, although the R^2^ values were small compared to DNA-based bacterial communities.

#### Taxonomic composition of bacterial communities

**Fig. 4:**
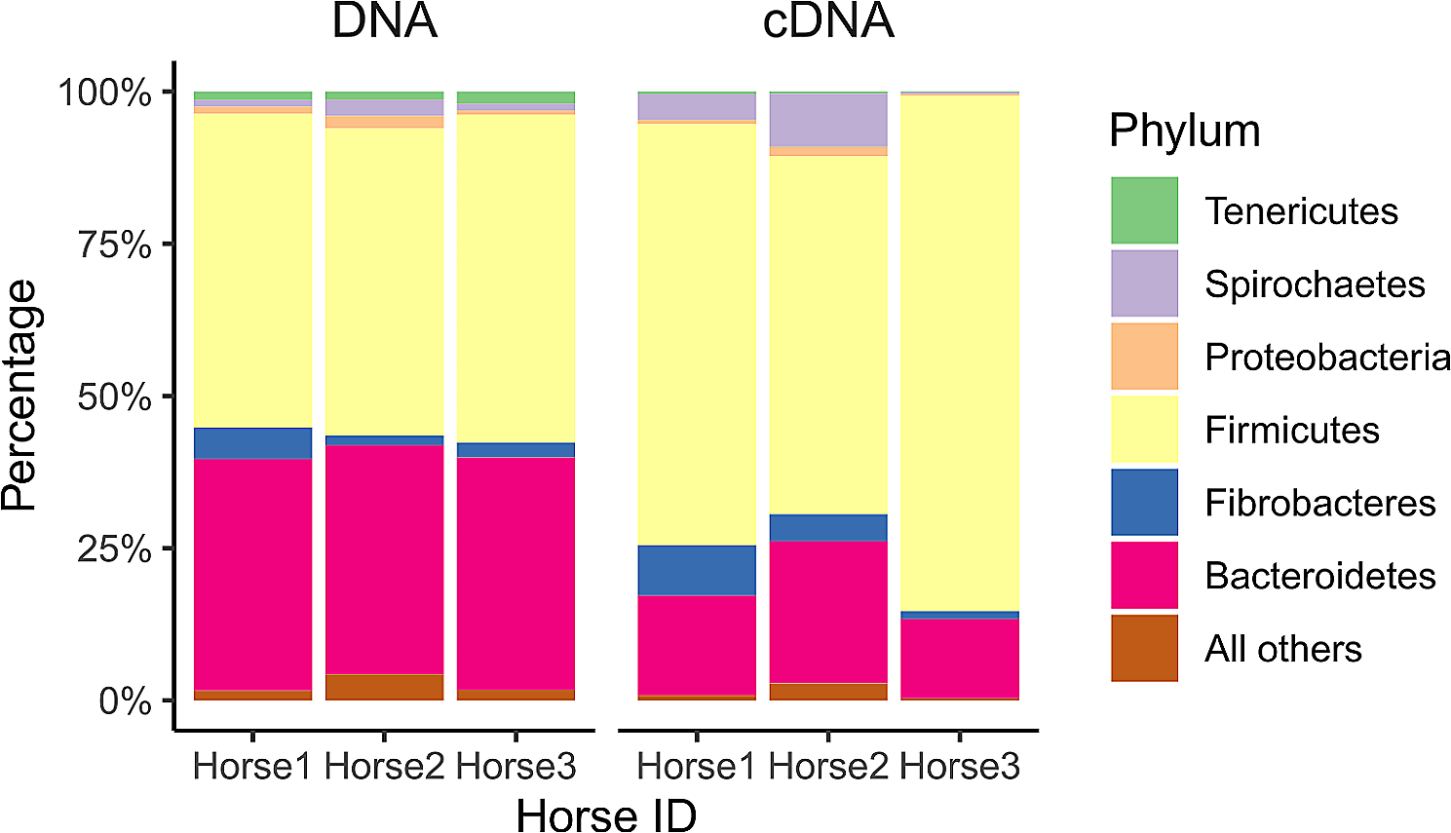
Mean relative abundance (percentage, %) at the phylum level for each individual horse. The figure is separated by extraction type (DNA on the left, cDNA on the right). For each horse (along the X axis), the height of the bar (Y axis) equates to the total percentage of all phyla present (100%), and each color represents a different phylum

The mean relative abundance of the major bacterial phyla for DNA and cDNA-based extraction is shown in Table 2, with mean relative abundance at each storage variable combination presented in Additional Table 3. Additionally, relative abundance for both DNA and cDNA-based extraction for individual horse is shown in Fig. 4. When comparing cDNA to DNA analysis in the fresh samples (saline cDNA, saline plus glycerol cDNA vs. saline DNA, saline plus glycerol DNA), the relative abundance of Firmicutes (70.42%, 71.39% vs. 51.85%, 52.09%), Fibrobacteres (4.78%, 4.52% vs. 2.31%, 3.83), and Spirochaetes (2.81% vs. 1.72%) increased, while those of Bacteroidetes (18.03%, 17.14%% vs. 38.95%, 36.94%) and Tenericutes (0.27%, 0.32% vs. 1.58%, 1.54%) decreased.

**Table 2:** Mean relative abundance of phyla (percentage, %)

	DNA			cDNA		
	Fresh^a^	Saline^b^	Saline plus glycerol^b^	Fresh^a^	Saline^b^	Saline plus glycerol^b^
Actinobacteria	0.80	1.08	0.76	0.37	0.57	0.42
Bacteroidetes	37.95	33.53	37.33	17.58	16.84	19.08
Fibrobacteres	3.07	1.06	2.94	4.65	2.48	4.79
Firmicutes	51.97	59.78	52.91	70.90	74.85	69.91
Proteobacteria	1.29	0.96	1.11	0.85	0.99	1.09
Spirochaetes	1.66	1.40	2.13	4.41	2.51	2.85
Tenericutes	1.56	0.98	1.47	0.29	0.34	0.40

Phyla with > 1% mean relative abundance at any storage time or condition.

^a^All fresh samples for both saline and saline plus glycerol.

^b^All frozen samples for both − 20 °C and − 80 °C.

At the genus level, a total of 62 genera were identified as commonly present populations with a cutoff of 75% presence across all samples (Additional Table 4a and 4b). All these genera showed significant differences between DNA and cDNA bacterial communities on day 0, the fresh sample, except for unclassified GMD14H09 within the Proteobacteria phylum (Additional Table 5). Within each extraction type, bacterial communities were compared by buffer and day (day 30, day 60 and day 90) to their respective controls at -20 °C and − 80 °C using glmer model with animal as the random effect.

**Fig. 5:**
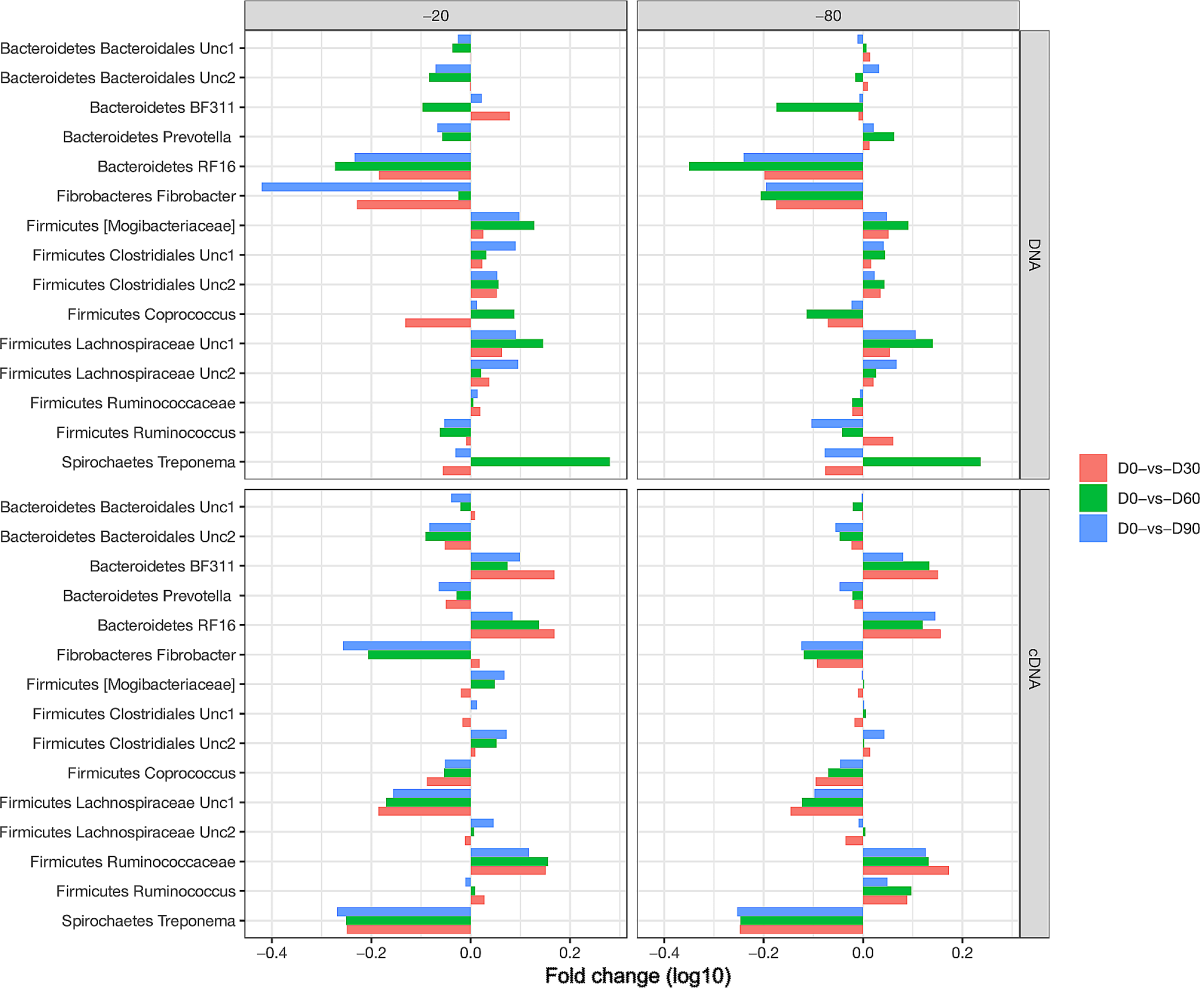
Fold change in relative abundance of genera common to both DNA-based and cDNA-based analysis. Included genera have a mean relative abundance of > 1%. Each pane depicts either DNA- based or cDNA- based analysis for a given storage temperature (-20 °C or -80 °C). The Y axis represents the fold change in relative abundance (log10). The X axis represents the individual genera, grouped by three colored bars which indicate the fold change at each day of frozen storage (D30, D60, D90) as compared to fresh (D0)

Within DNA-based bacterial communities (Additional Table 4a) the most abundant genera (> 1%) in the phylum Firmicutes were unclassified Clostridiales 1 (9%), unclassified Lachnospiraceae 1 and 2 (7.9 and 7.1, respectively), unclassified Ruminococcaceae (7.6%), unclassified Clostridiales 2 (4.7%), *RFN20* (2.5%), unclassified Mogibacteriaceae (2.1%), *Phascolarctobacterium* (1.8%), *Ruminococcus* (1.9%), *Coprococcus* (1.2%) and *Eubacterium*-like genera (1%) in the fresh samples. At either − 20 °C or -80 °C, Bacteroidetes lineages were generally decreased whereas Firmicutes were increased with storage when compared to fresh (Fig. 5). When compared to -80 °C, shifts in the relative abundance of lineages were more variable in -20 °C. While a majority of Firmicutes seemed to be fluctuating by day as well as buffer (Additional Table 6), some lineages, particularly *Christensenellaceae, Roseburia, Eubacterium, Oscillospira* and *Dorea*, were not affected by buffer. For the larger 1-liter aliquot, several Firmicutes genera showed changes with frozen storage, particularly with the saline plus glycerol buffer (Additional Fig. 4).

Among the Bacteroidetes, the second most abundant phylum, the most abundant genera in fresh samples were unclassified Bacteroidales (18.9%), unclassified RF16 (3.7%), Prevotella (2.9%), unclassified Bacteroidales 2 (2.8%), *BF311* (1.9%), *YRC22* (1.8%), *Paludibacter* (1.7%) and *CF231* (1.6%) (Additional Table 4a). Among these, *Paludibacter*, unclassified RF16, and unclassified S24-7 were the only genera that showed differences by buffer, day, and their interactions at both − 20 °C and − 80 °C. Notably, the unclassified lineages of Paraprevotellaceae showed differences by day only at -20 °C whereas the genera including *Prevotella*-like, *CF231*, *YRC22* and *Bacteroides* showed differences by day only at -80 °C. Further, unclassified Paraprevotellaceae, *BF311*, *CF231*, and unclassified Marinilabiaceae were not affected by buffer at either − 20 °C and − 80 °C. When examining the 1-liter storage size, Bacteroidetes lineages showed significant changes with frozen storage, particularly with saline plus glycerol buffer (Additional Fig. 4). Among the less dominant bacterial phyla of Fibrobacteres, the only commonly present genus *Fibrobacter* was reduced (*P* < 0.001) by buffer, day, and their interactions. Also, notable observations are the Actinobacteria and Tenericutes except unclassified Mycoplasmataceae that were not affected by buffer at either − 20 °C and − 80 °C while Proteobacteria, Fibrobacteres, and Spirochaetes except *Sphaerochaeta* showed fluctuations with buffer.

The cDNA-based bacterial communities (Table 2, Additional Table 3) were predominated by Firmicutes (> 70%) whereas Bacteroidetes comprised < 20% followed by Fibrobacter and Spirochaetes at 4.5% in fresh samples which is different to the profiles of DNA-based bacterial communities. Among Firmicutes, the most abundant genera (> 1%) in the phylum Firmicutes (Additional Table 4b) are unclassified Lachnospiraceae 1 at 14%, unclassified Clostridiales 1 (11%), unclassified Lachnospiraceae 2 (10%), unclassified Ruminococcaceae (9.8%), unclassified Clostridiales 2 (4.6%), *Ruminococcus* (4%), unclassified Mogibacteriaceae (2.9%), *Coprococcus* (2.5%), *Eubacterium*-like genera (1.6%) and *Phascolarctobacterium* (1.3%) in the fresh samples. Notably, there were more differences in the relative abundance of genera by buffer and day in cDNA communities (Additional Table 6), and these were consistent at -20 °C or -80 °C. For example, samples stored in saline in which relative abundance was increased by day of storage showed the same trend at both − 20 °C and − 80 °C, suggesting storage temperature did not have an effect, but storage buffer did. Compared to fresh, samples stored in saline had most genera within Firmicutes increase with storage day; however, the relative abundance of most genera within Firmicutes was stable when samples were stored in saline plus glycerol at both − 20 °C and − 80 °C.

In contrast, genera within Bacteroidetes were generally reduced with frozen storage day compared to fresh at both storage temperatures, irrespective of buffer. *Fibrobacter* genus had a greater contribution in cDNA compared to DNA communities in fresh samples, and the relative abundance was almost halved in saline at both temperatures but remained stable in saline plus glycerol at both storage temperatures. Among Spirochaetes, *Treponema* had the highest relative abundance in cDNA communities; however, with storage the relative abundance was halved in all storage days compared to fresh, irrespective of different buffers and temperatures.

## Discussion

The results of this study support our hypothesis that when assessing bacterial populations, both at the community level and individual taxa, there were marked differences between DNA and cDNA-based components, irrespective of fresh or stored samples. Further, shifts within and among bacterial communities were noticeable with length of storage in both DNA and cDNA communities; however, storage conditions such as temperature and buffer had interactions with length of storage which in turn induced distinct shifts in equine fecal DNA and cDNA bacterial communities. Findings of this study provided novel information on results obtained when assessing total and potentially metabolically active bacterial populations within the gastrointestinal tract of equines. It also sets the stage for making recommendations on the best storage conditions to reduce perturbations induced during storage and preserve viability of bacterial populations.

The first most significant finding of this study is that changes induced in fecal microbiota with different storage conditions are marginal compared to the variations between horses. Several studies from human as well as diverse animal species have reported the inter-animal differences in microbial fingerprints [28, 35–39]. The three mares in this study, that differed in age and breed characteristics yet shared management practices, had differences in their bacterial populations. One individual horse was found to have a high abundance of Firmicutes in fresh feces as compared to the other two horses in the study. This horse was the oldest of the three mares (14 years, as compared to 8 and 6 years of age). Despite these differences between horses, Firmicutes was the most dominant phylum followed by Bacteroidetes with both DNA-based and cDNA-based analysis. However, the relative abundance of these two phyla varied by horse, extraction type (DNA or RNA), and storage conditions. The equine fecal microbiota is dominated by two phyla, Firmicutes and Bacteroidetes, which based on data from healthy horses have relative abundances of 14 to 36% and 37 to 68%, respectively [14–16, 28]. The other major phyla of the equine bacterial microbiota include Proteobacteria (0.8 to 10.2%), Spirochaetes (2 to 6%), and Fibrobacteres (1.5 to 4.2%) [14–16, 28, 40].

The second most notable finding of this study was the difference in community composition observed when using the two separate extraction methods (DNA and cDNA). While Firmicutes and Bacteroidetes together comprised the dominant bacterial populations across the two fractions, lineages of Firmicutes had a greater contribution with Bacteroidetes reduced to almost half their populations in cDNA based communities. Also notable is the contribution of *Fibrobacter* and *Treponema* which appear to have insignificant roles in DNA-based communities but have greater contributions in cDNA, indicating that certain populations may have a greater metabolic footprint than what can be gleaned from gene copies. These discrepancies between total and potentially metabolically active populations in different phyla and genera could highlight that some taxonomic groups, although they have lower or higher gene copies, may have the opposite effect in terms of their functional contributions. Such findings have also been reported in the ruminant microbiome, with Bacteroidetes having lower contribution and Proteobacteria higher contribution in cDNA-based communities [32, 41]. A new observation was the greater contribution of *Treponema* in metabolically active fraction (cDNA). Both *Fibrobacter* and *Treponema* were reported to be positively associated with, and found to in greater abundance in, horses with colic while also being reduced in recovery [15]. Although diet is a major driver to induce changes in bacterial populations, and these populations are lower in diets with less fiber, the role of these bacteria in health and disease requires further investigation.

To our knowledge, the application of cDNA-based sequencing has not been applied to equine feces previously, although it has been used in other species, such as cattle to assess the rumen microbiome [42]. RNA is utilized as a marker of the potential of organisms for metabolic activity because only functional cells produce RNA as part of normal cell function and reproduction [33, 34]. Metabolic potential of bacteria was assessed in this study using cDNA-based 16 S rRNA sequencing. Standard metagenomic analysis of DNA, although providing a comprehensive view of the bacteria present within a sample, does not directly assess activity or potential for metabolic activity of the members of the microbiome, because it cannot differentiate genes from live cells, live but inactive cells, or dead cells [33, 43, 44]. It is for these reasons that cDNA-based sequencing was chosen to assess the potential for metabolic activity in equine fecal transplant preparations, since activity of transplanted bacteria is important for clinical effect in the patient. Previous work has demonstrated that RNA-based sequencing is superior for detecting live bacterial cells, and that RNA concentrations correlate with metabolic activity [33, 45]. RNA sequencing does not allow for a direct evaluation of bacterial growth or current level of activity, but rather is a marker of protein synthesis potential (found in both dormant and active cells). Several studies have used RNA-based 16 S rRNA sequencing to evaluate for viable bacteria in both human and animal samples [32, 44, 46]. Since cDNA-based sequencing is currently less widely used than DNA-based analysis, we performed paired analysis of both DNA and cDNA to allow for assessment of bacterial communities using both methods within the same equine fecal samples.

RNA sequencing can also be used in metatranscriptomic analysis to investigate the presence of functional metabolic pathways, host-microbe interactions, and other more complex population dynamics. The fast degradation of RNA complicates sample handling and analysis [47]. Fast collection, preservation with ice or liquid nitrogen, or the addition of RNAse inhibitors, can help ensure preservation of the nucleic acid material of interest [47]. In the present study, we used RNAse inhibitors and immediately froze samples intended for cDNA-based analysis after slurry preparation to ensure preservation of RNA. Preserving the samples in trizol or RNALater has been traditionally employed to preserve RNA integrity; however, our attempts to store equine feces in trizol did not yield adequate results. Nevertheless, addition of inhibitors followed by immediate freezing enabled us to extract decent quantities of RNA and compare the bacterial profiles of cDNA with those of DNA. Previous studies evaluating the effect of storage on equine fecal microbiota or fecal transplant material microbiota have focused mainly on the effect of storage temperature on the microbiota. Ambient temperature storage consistently shows a significant change in diversity and population composition over time across studies [48, 49]. One previous study of equine fecal microbiota showed both − 80 °C and − 20° C storage preserved diversity and bacterial composition for up to several years [50], while a separate study found a decrease in viability of gram-negative bacteria after processing and storage of an equine fecal transplant material at -20° C within weeks [30]. All previous studies assessed either total bacterial population with DNA-based sequencing, or viability using a culture-based method or PMA treatment of samples followed by DNA-based sequencing.

In our study, we investigated the effects of storage conditions including temperature at which samples are stored (-20 °C and − 80 °C) to represent shallow or deep-freezing techniques, buffers such as saline or saline plus glycerol, and length of frozen storage. While differences between horses were obvious, when variation between animals was incorporated into the models, the effects of storage conditions were determined, albeit small compared to differences between horses, and with the inherent limitation of small sample size (*n* = 3). While the study was ambitious to investigate several storage conditions, several of our findings correlate with previous work in other species and lay the foundation for generating several hypotheses that need further investigations.

Using glmer model for alpha diversity and individual taxa with animal as random effect enabled us to unravel new information that requires further validations. Within DNA communities, length of storage has a significant impact on the bacterial community profiles. A notable observation in both alpha diversity and individual taxa was the deviation of day 60 from day 30 and day 90. Most lineages of Bacteroidetes had an increased relative abundance (making up a larger percentage of the overall community) at day 30, had a sharp decline at day 60 and then increased relative abundance by day 90. Concurrently, lineages of Firmicutes had increased relative abundance at day 30, had a sharp rise or decline at day 60, with the opposite pattern at day 90. *Fibrobacter* decreased in relative abundance at day 30, increased in relative abundance on day 60 and then declined on day 90. Although these patterns were observed across both buffers, saline plus glycerol had less fluctuations compared to saline only preserved samples. Compared to -20 °C, shifts were less intense in -80 °C, with marginal changes in saline plus glycerol at -80 °C. The spurious fluctuations at day 60 in DNA communities across both temperatures and buffers, is interesting, although reasons remain unknown. Such fluctuations were also noted by Ma et al. when the authors compared fecal microbial profiles for short periods (1 and 2 weeks) and longer-term storage such as 6 months compared to fresh [51]. There could be several factors affecting such patterns including possible changes when the sample must transition from room temperature to frozen condition, during refrigeration, and thawing conditions which can affect the Gram-positive bacteria. Irrespective of DNA or cDNA, storing samples increased the relative abundance of Firmicutes and *Fibrobacter* which are Gram positive bacteria and associated with cellulose digestion. A decrease in the relative abundance of Bacteroidetes and an increase in that of Firmicutes with time in equine fecal microbiota has been reported which corroborates with findings of this study [40]. The sensitivity of Bacteroidetes lineages as the first to fall and early to recover with external perturbation has been reported previously, and therefore, the reductions in relative abundance of DNA-based Bacteroidetes lineages with storage as observed in this study is not surprising [32, 52, 53]. Further investigations should investigate the effect of spiking with mock populations representing Bacteroidetes, Firmicutes, Fibrobacter and Treponema to assess their tenacity to reacting with different storage conditions.

Another interesting observation was the resilience of Actinobacteria members, *Roseburia*, *Oscillospira*, *Christensenellaceae*, *Eubacterium*, and *Dorea*, that were not affected by buffers at both temperatures within the DNA component. A common function that ties all these bacteria is their ability to produce butyrate. Most butyrate producers can participate in biohydrogenation and this enables them to protect the colonic microbiome from stress induced by polyunsaturated fatty acids [54]. Whether these bacteria continue to participate in biohydrogenation, and if the resulting metabolites naturally protect these bacteria from abrasions induced by buffer and freezing interactions, remains to be investigated in the future.

Finally, compared to fluxes induced by buffer, temperature, and day interactions in DNA-based communities, cDNA populations experienced less intense fluctuations across different storage conditions. Either at the community level or individual taxa, variations in relative abundances of individual taxa were less across days at both temperatures. Clearly, saline was not an ideal buffer to preserve metabolically active populations, as changes were noted by buffer in saline preserved samples at both storage temperatures, However, saline plus glycerol stored samples were relatively stable across both temperatures, with even less variations in the relative abundances of individual taxanoted at -80 °C. The use of cryoprotectants such as glycerol help preserve organisms and tissues by lowering the freezing point of water to prevent crystallization and mechanical damage [55]. Glycerol, which forms hydrogen bonds with water molecules and can penetrate both the cell wall and cell membrane, is widely used for preservation in microbiome research, and is recommended for the preservation of fecal microbiota transplant material in human medicine [55, 56]. The results in individual studies are somewhat mixed, with some finding improved preservation without any buffer [57, 58]. However, in our study the use of saline plus glycerol overall appeared superior to saline alone for preserving the potentially metabolically active bacterial populations. Interestingly, no obvious differences were noted from fresh samples at either storage temperature (-20 °C–− 80 °C). In field conditions where access to a -80 °C is not always possible, archiving samples at -20 °C may be suggested.

Most of the sample volumes used in this study were small aliquots (1.5 ml volume). The 1-liter sized samples were included to mimic a volume that would be clinically useful to store, with only DNA-based analysis at day 90 of -80 °C frozen storage analyzed. Results of the analysis showed a difference in alpha and beta diversity with frozen storage, more dramatic than that seen for the smaller aliquot sizes. Conclusions about the ideal storage volume cannot be made based on the current data, due to the limited number of samples prepared. Previous studies evaluating the effect of storage on equine fecal transplant material have utilized relatively small aliquot sizes, with no evaluation of different storage volumes on the microbiota [30, 31].

The major limitation of this study was the small number of horses used for analysis. As discussed above, individual horse influenced several different measures, and the small sample size could have increased the potential for Type II error. Future studies with greater numbers of horses are needed to determine the best storage conditions for the general equine population. Additionally, we performed qualitative evaluation of bacterial populations (relative abundance) based on 16s analysis, and therefore do not have quantitative data for changes in populations between extraction method or storage variables. Taxonomic composition changes using relative abundance are expressed as a percentage of all taxa present, and therefore increases or decreases in relative abundance numbers indicate a corresponding decrease or increase in another bacterial taxonomic group. Finally, the ideal storage volume was not assessed in this study, with most samples being 1.5 ml in volume. As discussed, the larger 1-liter aliquot size showed significant differences when comparing frozen to fresh samples.

## Conclusion

Assessment of potential bacterial metabolic activity via cDNA-based analysis is feasible for the equine fecal microbiome and allows for effective population analysis when compared to routine DNA-based sequencing. Major differences were seen in relative abundance of specific bacterial populations when comparing DNA-based and cDNA-based analysis, which has not been previously evaluated in the equine fecal microbiota. Major differences based on individual horse were seen particularly in markers of alpha and beta diversity, and overall, the findings of differences between individuals highlight the need for larger sample sizes in future studies.

Our study aimed to evaluate the effect of multiple storage variables on the composition of a stored frozen fecal transplant material. Based on our findings, the potentially metabolically active component of the equine fecal microbiome was overall minimally affected by frozen storage, with the use of a saline plus glycerol buffer appearing protective for many bacteria at the storage temperatures and length of storage evaluated here. Future studies should utilize larger population sizes and investigate how these metabolically active populations change with and can be better protected in storage, to provide a more effective FMT product for clinical use. Investigation into how best to preserve larger aliquot sizes will be necessary to allow for clinical application.

## Methods

### Animals

Three horses from a university teaching herd were used in the study (Table 3). Horses chosen were confirmed to have no recent history of colic within the last year or medication administration within the last 30 days. All horses were housed continuously on pasture with supplementation of timothy hay.

**Table 3:** Individual animal characteristics for the three horses

Horse	Gender	Breed	Age (years)	Weight (kg)
Horse 1	Mare	Friesian	8	550
Horse 2	Mare	Appaloosa	6	520
Horse 3	Mare	Warmblood	14	580

### Sample collection

Horses were sedated with intravenous xylazine (0.3–0.5 mg/kg; XylaMed, VetOne^®^, MWI Animal Health, Boise, ID, USA) and manually restrained in standing stocks prior to collection of a fecal sample via transrectal palpation. Approximately 600 to 800 g of fresh feces was collected from each horse, and the feces was kept in the rectal sleeve used to collect the sample, with air pushed from the sleeve and the end of the sleeve tied. Immediately following collection, all fecal samples were transported to the laboratory on ice for slurry preparation and arrived at the lab within 10 min of collection.

### Fecal slurry preparation and storage

**Fig. 6:**
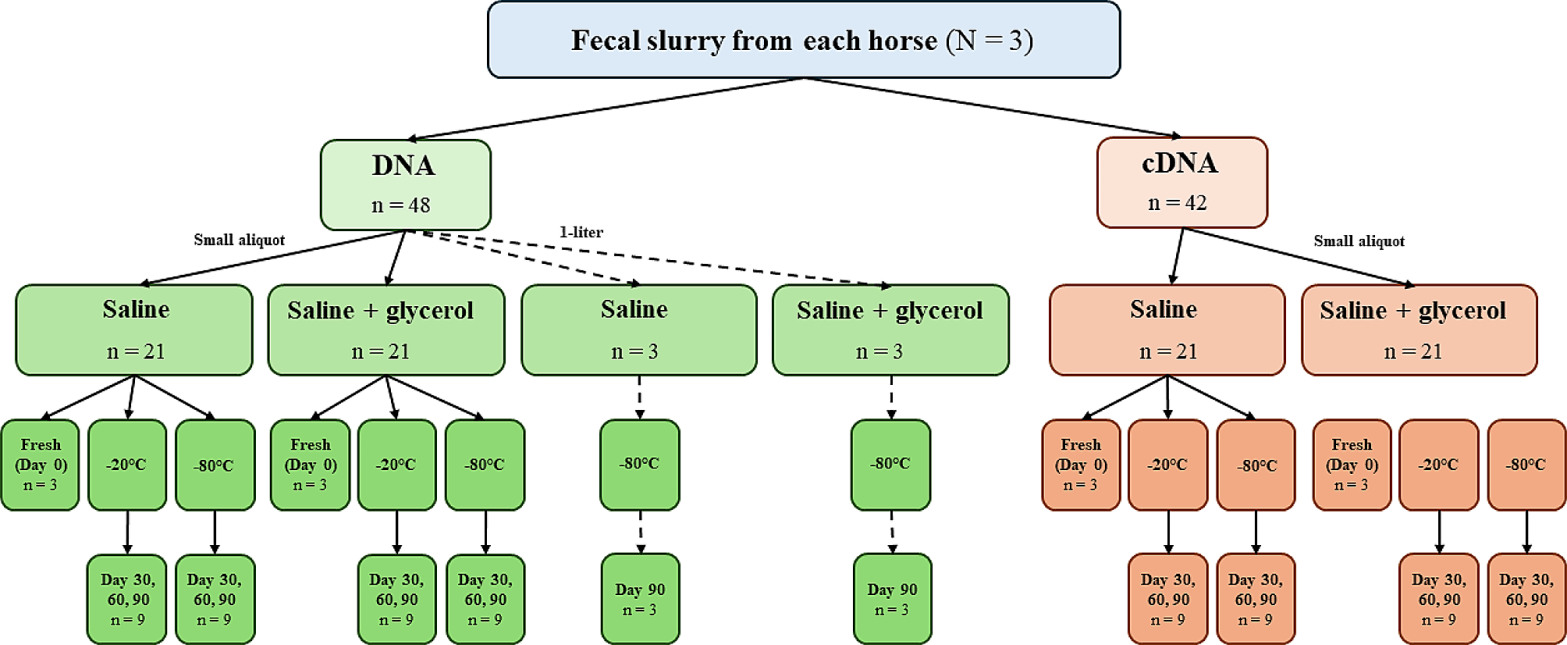
Overview of sample preparation and the combination of storage variables analyzed. Sample size for each of the storage variable combinations is denoted by “n=”. Small aliquots are represented by solid lines, while 1-liter storage size (DNA only) is represented by dotted lines. For all storage sizes, DNA extraction was performed on 400 µl of slurry and RNA extraction was performed on 300 µl of slurry. RNA was reverse transcribed to cDNA for PCR amplification and subsequent sequencing, with DNA directly PCR amplified and sequenced. Both DNA-based and cDNA-based analysis was performed on fresh samples (Day 0) and frozen samples at both storage temperatures (-20 °C and − 80°Cat Day 30, 60, and 90 of frozen storage. For the aliquots stored in 1-liter volumes these were analyzed with DNA-based analysis only after 90 days of storage at -80 °C

Sample preparation is summarized in Fig. 6. The three horses were sampled in a sequential manner. Each horse was sampled and processed as detailed below. The RNA extraction procedure took approximately 2 h on fresh samples, and therefore Horse 2 was sampled after the completion of RNA extraction from Horse 1 fresh samples. Similarly, Horse 3 was sampled after Horse 2 fresh samples were processed for RNA extraction. However, DNA from all fresh samples were performed after completion of sampling and processing of all 3 horses. The fresh samples were kept at 4^o^C until DNA extraction, which was approximately 6 h after collection.

Preparation was performed in two 2.4 L capacity household blenders that had been purged with carbon dioxide (CO_2_) for 5 min, with one blender used for the saline only slurry and one used for the saline plus glycerol slurry. All equipment was rinsed with an RNase inhibitor solution before and between sample processing to decrease RNA degradation (ELIMINase^®^, Decon Labs, Inc., King of Prussia, PA, USA). Blenders were cleaned thoroughly between each horse, with the same two blenders used for all three horses.

To prepare the saline only slurry, 62.5 g of fresh stool and 187.5 mL of sterile saline (0.9% sodium chloride irrigation USP; B. Braun Medical Inc., Irvine, CA, USA) were added to one blender and blended at high speed for approximately 1 min. To prepare the saline plus glycerol slurry, for a final dilution of 10% glycerol, 62.5 g of fresh stool, 162.5 mL of saline, and 25 mL of glycerol (85% glycerol solution; Honeywell Riedel-de Haën, Muskegon, MI, USA) were added to the second blender and blended at high speed for approximately 1 min; this was done simultaneously with the saline-only slurry [59]. At the completion of blending, the individual samples were then separated into smaller aliquots for storage (frozen samples) or analysis (fresh samples). First, 300 µL of each of the slurries were pipetted in duplicate into a bead-beating tube (Lysing Matrix E 2-mL tubes; MP Biomedicals, Irvine, CA, USA) and mRNA immediately extracted as described below (fresh, day 0 cDNA sample). mRNA extraction was completed before beginning slurry and aliquot preparation on the next horse. Next, 1.5 mL of each slurry was aliquoted into 2-mL Eppendorf tubes for storage in either a -20 °C or -80 °C freezer for analysis at day 3, 7, 14, 30, 60, 90 (cDNA) or day 30, 60, 90 (DNA). A 1.5 ml slurry sample was stored at 4 °C for up to 4 h for DNA extraction later the same day (fresh, day 0 DNA sample).

After small aliquots were prepared and stored at respective temperatures, 1-liter slurries were prepared to evaluate the effect of storage on larger storage volumes. To prepare the 1-liter slurries, an additional 200 g of stool and 600 mL of saline (for saline only slurry) or 200 g of stool, 520 mL of saline, and 80 mL of glycerol (for saline plus glycerol slurry) were added to each blender in two batches, blended for approximately 1 min per batch, poured into a 1-liter Nalgene bottle (Thermo Fisher Scientific, Waltham, MA, USA). For fresh (Day 0) sample a 400 µl volume of slurry was used for DNA extraction at this time (described below), and the remainder was stored in a -80 °C freezer until DNA extraction on day 90. The final number of fecal aliquots analyzed for all storage variable combinations (after removal of one frozen sample due to insufficient reads) consisted of 47 for DNA-based and 78 for cDNA-based analysis. Of these, there were 6 fresh samples each for both DNA-based and cDNA-based analysis, with 41 frozen samples for DNA-based and 72 frozen samples for cDNA-based analysis.

While the RNA from fresh samples of each horse was processed immediately to prevent RNA degradation, DNA extraction from fresh samples awaited until all samples were processed and archived at the above-described storage conditions. The stored samples were retrieved from the respective temperatures after the specified stored period had been completed and left to thaw on the benchtop at room temperature. As the volume was small, samples were thawed in approximately 15 min, after which the samples were extracted for DNA and RNA immediately. However, for the 1-liter bottles, as the volume is more, the samples were thawed at 4^o^C before DNA extraction which took approximately 30 min.

### Nucleic acid extraction, amplification, and sequencing

DNA was extracted from 400 µL of slurry using the “repeated bead beating and column” (RBB + C) method based on the procedure described in Yu and Morrison [60] followed by extraction with a commercial kit (QIAmp Fast DNA Stool Mini Kit; Qiagen Sciences, Germantown, MD, USA). RNA was extracted from 300 µL of slurry using the RNeasy PowerPlant Kit (Qiagen Sciences) with minor modifications. RNA was reverse transcribed to cDNA using the SuperScript VILO cDNA Synthesis Kit (Invitrogen, Carlsbad, CA, USA) according to the manufacturer’s protocol. DNA was extracted on day 0 (fresh), day 30, day 60, and day 90. RNA was extracted on day 0 (fresh), day 3, day 7, day 14, day 30, day 60, and day 90 and then reverse transcribed to cDNA. More sampling time points were evaluated for cDNA as the main purpose of this study was to assess the effect of frozen storage on potentially metabolically active bacterial populations. Additionally, DNA only (not RNA) was extracted from the 1 L slurry samples at day 90, using a 400 µl volume of slurry for extraction. For each DNA and cDNA sample, the V1-V2 region of the bacterial 16 S rRNA gene was PCR-amplified in triplicate using the bacterial-specific primers F27 (5′-AGAGTTTGATCCTGGCTCAG-3′) and R338 (5′-TGCTGCCTCCCGTAGGAGT-3′) barcoded with a unique 12-base error-correcting Golay code for multiplexing as described previously [61]. Polymerase chain reaction was performed in triplicate using the Accuprime Taq DNA Polymerase System (Invitrogen). The thermal cycling conditions involved an initial denaturing step at 95° C for 5 min followed by 20 cycles (denaturing at 95° C for 30 s, annealing at 56° C for 30 s, extension at 72° C for 90 s) and a final extension step at 72° C for 8 min. The amplicons from each DNA sample were combined and each library was added to a pool in equimolar concentration. Two DNA extraction blanks, two cDNA extraction blanks, and two PCR amplification blanks were included in the final pool. The final pool was bead purified using Beckman Coulter Agencourt AMPure XP Beads (Beckman Coulter, Brea, CA, USA). Sequencing was performed at the PennCHOP Microbiome Core, University of Pennsylvania, using the Illumina MiSeq platform.

### Bioinformatics

The bacterial 16S rRNA sequencing data was processed through the QIIME2 (2020.6) pipeline as described previously [52, 62]. Briefly, raw reads were de-multiplexed and amplicon sequence variants (ASV) were obtained using the Divisive Amplicon Denoising Algorithm 2 (DADA2) plugin available in QIIME2 [63]. The representative ASV were subjected to multiple sequencing alignment using MAFFT and a mid-point rooted tree was then generated using FastTree 2 [64, 65]. A pre-trained Naive Bayes classifier trained on the Greengenes database (v13.8) for the 16S rRNA gene spanning the V1-V2 region was used for taxonomic classification [66]. Alpha diversity (observed ASV and Shannon diversity) and. Beta diversity was assessed using both weighted and unweighted UniFrac distances. Weighted UniFrac considers the relative abundance of microbial taxa and their phylogenetic relationships between samples, while unweighted UniFrac only considers the presence or absence of microbial taxa and their phylogenetic relationships between samples. These analyses were conducted utilizing the ‘qiime diversity’ plugin following rarefaction of samples to 5,898 reads per sample.

### Statistical analysis

All the statistical analyses were conducted in R software (R version 4.3.1, R Core Team 2023). A mixed linear model was used to analyze alpha diversity metrics (observed ASV and Shannon diversity) using the lmerTest package, while a Generalized Linear Mixed Model (GLMM) was applied to individual taxonomy data (genus level) using the lme4 package. The statistical model was specified as follows (Eq. 1).

Eq 1 $${\text{Y}} = \beta {\text{0}} + \beta 1.Buffer + \beta 2.Day + \beta 3.\left( {Buffer*Day} \right) + \mu + \in$$

where Y represents the response variable (alpha diversity or individual taxonomic relative abundance), β0 is the overall intercept, β1​ is the coefficient for the buffer effect (saline, saline plus glycerol), β2​ is the coefficient for the Day effect (0, 30, 60, and 90), β3​ is the coefficient for the interaction between Buffer and Day, µ represents the random effects of horse, and ɛ is the residual error.

For beta diversity matrices, a multivariate nonparametric permutational multivariate ANOVA (PERMANOVA) test was conducted using the vegan package in R [67]. We used the same statistical model as described in Eq. 1. The model included weighted or unweighted matrices as the response variable, with 999 permutations, and was blocked by horse (strata = Horse ID).

For all diversity indices and taxonomic composition analyses, fresh samples were compared to samples from days 30, 60, and 90 using the statistical model described in Eq. 1. These analyses were conducted separately for the two temperatures (-20 °C and − 80 °C) within DNA and RNA. For the frozen 1-liter storage size, comparisons were made directly to the same buffer at the fresh time point for DNA extraction only.

Taxonomic read counts were normalized to proportions before analysis. P-values were adjusted for multiple comparisons using the False Discovery Rate (FDR) procedure and presented as relative abundance (percentage) as mean ± standard error of the mean (SEM). Fold changes were calculated as the ratio of the difference between day 30 to fresh, day 60 to fresh, and day 90 to fresh, and these ratios were log2 transformed. Analyses were conducted separately for the two temperatures (-20 °C and − 80 °C) within both DNA and RNA. A P-value of less than or equal to 0.05 was used to define significance. A P-value between 0.05 and 0.1 was used to denote trend.

### Electronic supplementary material

Below is the link to the electronic supplementary material.

Additional Dataset 1: Relative abundance data for individual sample Description: Relative abundance data for each individual fecal sample at both the phylum and genus level

Additional Fig. 1: Measurement of bacterial community composition (beta diversity) for analysis type (DNA vs. cDNA) and horse Description: The principal coordinate analysis (PCoA) plot shows unweighted UniFrac distances between samples, with samples that are more similar located closer to one another. Each data point represents an individual slurry sample. Differences in beta diversity were seen between extraction type and individual horses, as observed by the clustering of each horse within extraction type, with more variation within each horse for DNA-based analysis than cDNA-based analysis

Additional Fig. 2: Measurement of bacterial community composition (beta diversity) for the storage variables day and temperature for DNA (A, C) and cDNA (B, D) extraction. Description: The principal coordinate analysis (PCoA) plot shows weighted UniFrac distances between samples, with samples that are more similar located closer to one another. Each data point represents an individual slurry sample. For this study, there was an effect of individual horse for all variables within both DNA and cDNA analysis. Day of storage did not show significant similarities for bacterial community (A, B). Fresh samples were similar in community composition, with less similarity among frozen samples (C, D)

Additional Fig. 3: Measurement of bacterial community composition (beta diversity) for individual storage variables Description: The principal coordinate analysis (PCoA) plot shows unweighted UniFrac distances between samples, with samples that are more similar located closer to one another. Each data point represents an individual slurry sample. For this study, there was an effect of individual horse for all variables within both DNA and cDNA analysis (A-F). Neither day of storage (A, B) nor buffer type (C, D) showed significant similarities for bacterial community. Fresh samples were more similar to each other in community composition than matched frozen samples (E, F)

Additional Fig. 4: Fold change in the relative abundance of genera common to both DNA-based and cDNA-based analysis for 1-liter storage size Description: Included genera have a mean relative abundance of > 1%. The 1-liter aliquot size was only stored at -80 °C and only underwent DNA-based analysis. The Y axis represents the fold change in relative abundance (log10). The X axis represents the individual genera, grouped by two colored bars which indicate the fold change for each buffer type (saline or saline plus glycerol)

Additional Table 1: Effects of storage variables and their interactions on alpha diversity indices within each frozen storage temperature and extraction type

Additional Table 2: Effects of individual storage variables on beta diversity patterns within each frozen storage temperature and extraction type

Additional Table 3: Mean relative abundance of phyla (percentage, %) depicted by day and temperature for each buffer in DNA and cDNA

Additional Table 4a: Mean relative abundance (percentage, %) of common genera between DNA-based and cDNA-based analysis, DNA-analysis

Additional Table 4b: Mean relative abundance (percentage, %) of common genera between DNA-based and cDNA-based analysis, cDNA-analysis

Additional Table 5: Difference of relative abundance for genera common to both DNA-based and cDNA-based analysis at day 0 (fresh sample)

Additional Table 6: Effect of storage variables and their interactions on mean relative abundance of common genera between DNA-based and cDNA-based analysis within each frozen storage temperature

## Data Availability

The fecal microbiome sequences have been deposited in the NCBI database under BioProject accession number PRJNA856622. The raw data sequencing has been deposited in NCBI accession number Ln 586-Ln587.

## Abbreviations

CDI: *Clostridiodes difficile* infection
FMT: Fecal microbiota transplant
IBD: Inflammatory bowel disease
PCoA: Principal coordinate analysis
