# Supplementary figures and images for: Assessment of fecal bacterial viability and diversity in fresh and frozen fecal microbiota transplant (FMT) product in horses
Source: BMC Vet Res. 2024 Jul 10;20:306. doi: 10.1186/s12917-024-04166-w (PMC11234551; doi:10.1186/s12917-024-04166-w)

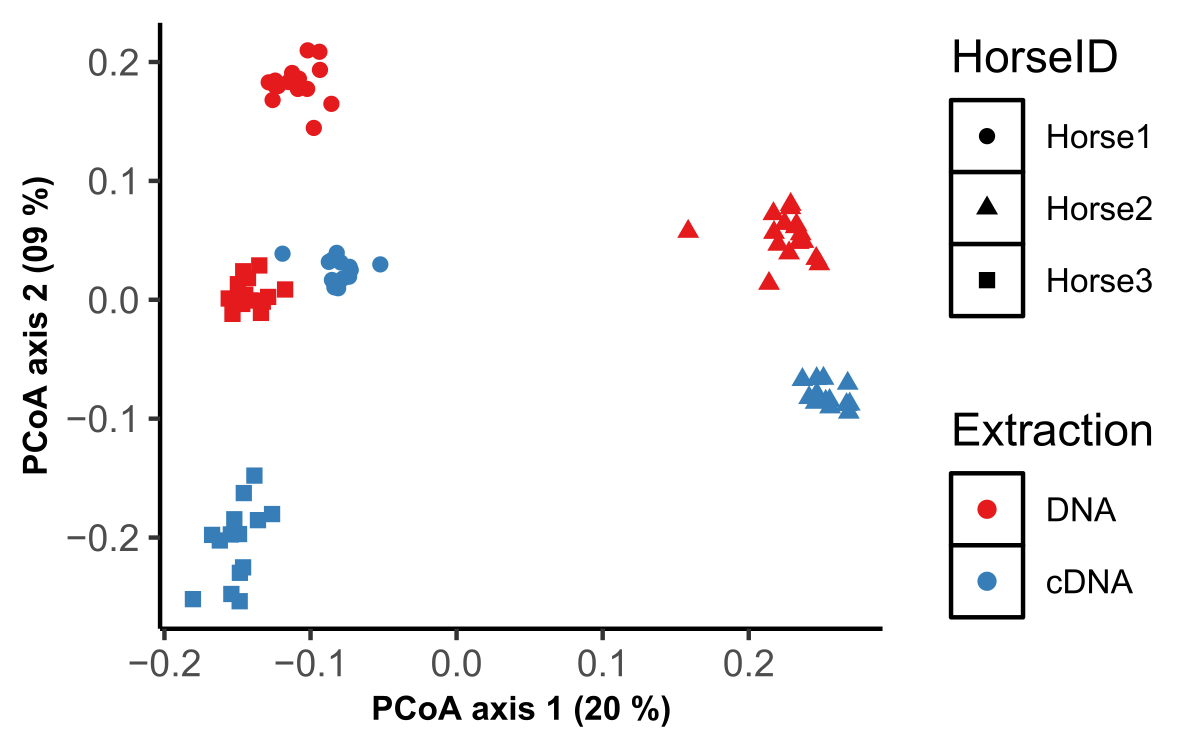

Supplement: Supplementary file 2 — Additional Fig. 1: Measurement of bacterial community composition (beta diversity) for analysis type (DNA vs. cDNA) and horse Description: The principal coordinate analysis (PCoA) plot shows unweighted UniFrac distances between samples, with samples that are more similar located closer to one another. Each data point represents an individual slurry sample. Differences in beta diversity were seen between extraction type and individual horses, as observed by the clustering of each horse within extraction type, with more variation within each horse for DNA-based analysis than cDNA-based analysis [file 12917_2024_4166_MOESM2_ESM.tif]

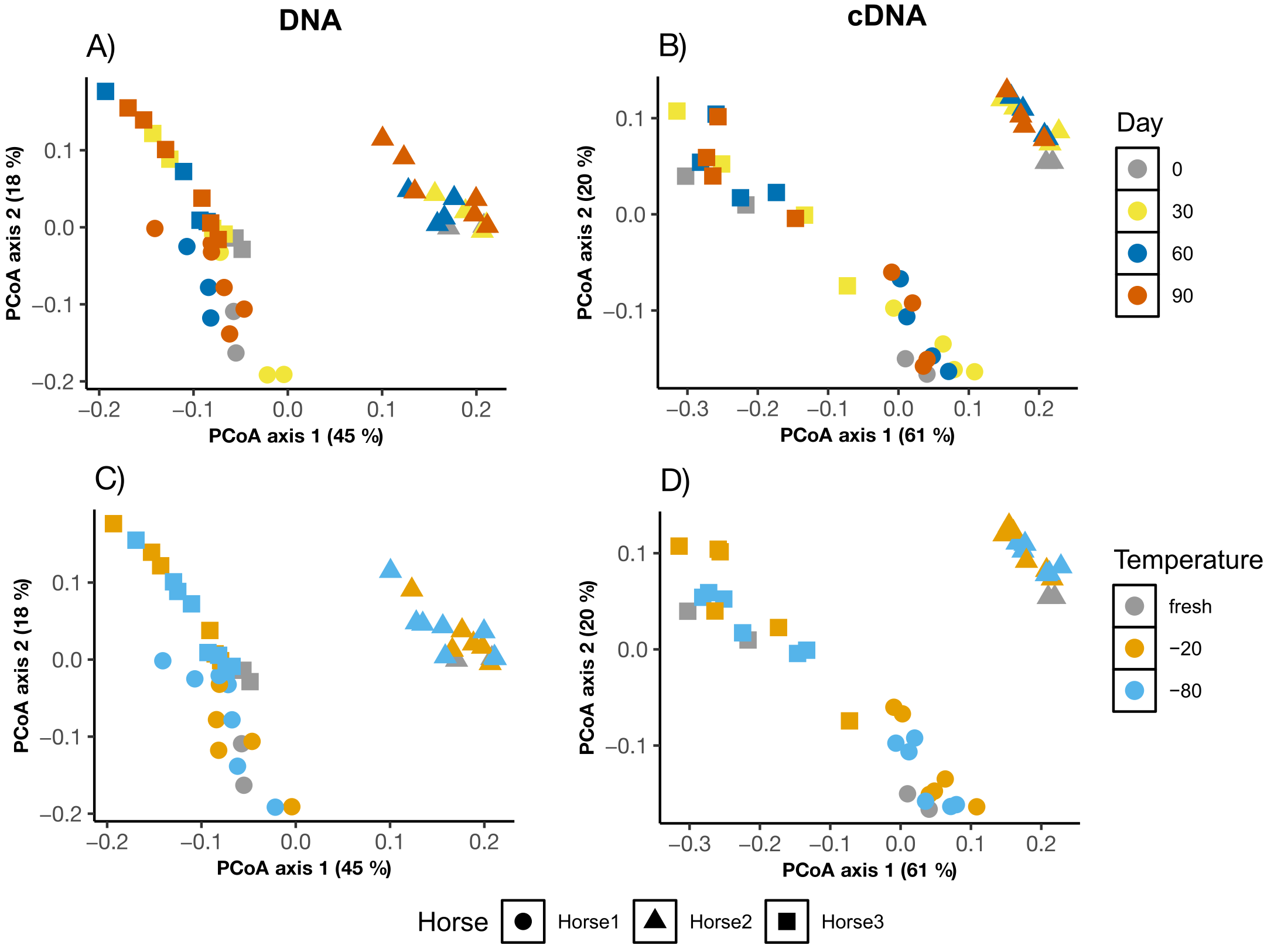

Supplement: Supplementary file 3 — Additional Fig. 2: Measurement of bacterial community composition (beta diversity) for the storage variables day and temperature for DNA (A, C) and cDNA (B, D) extraction. Description: The principal coordinate analysis (PCoA) plot shows weighted UniFrac distances between samples, with samples that are more similar located closer to one another. Each data point represents an individual slurry sample. For this study, there was an effect of individual horse for all variables within both DNA and cDNA analysis. Day of storage did not show significant similarities for bacterial community (A, B). Fresh samples were similar in community composition, with less similarity among frozen samples (C, D) [file 12917_2024_4166_MOESM3_ESM.tif]

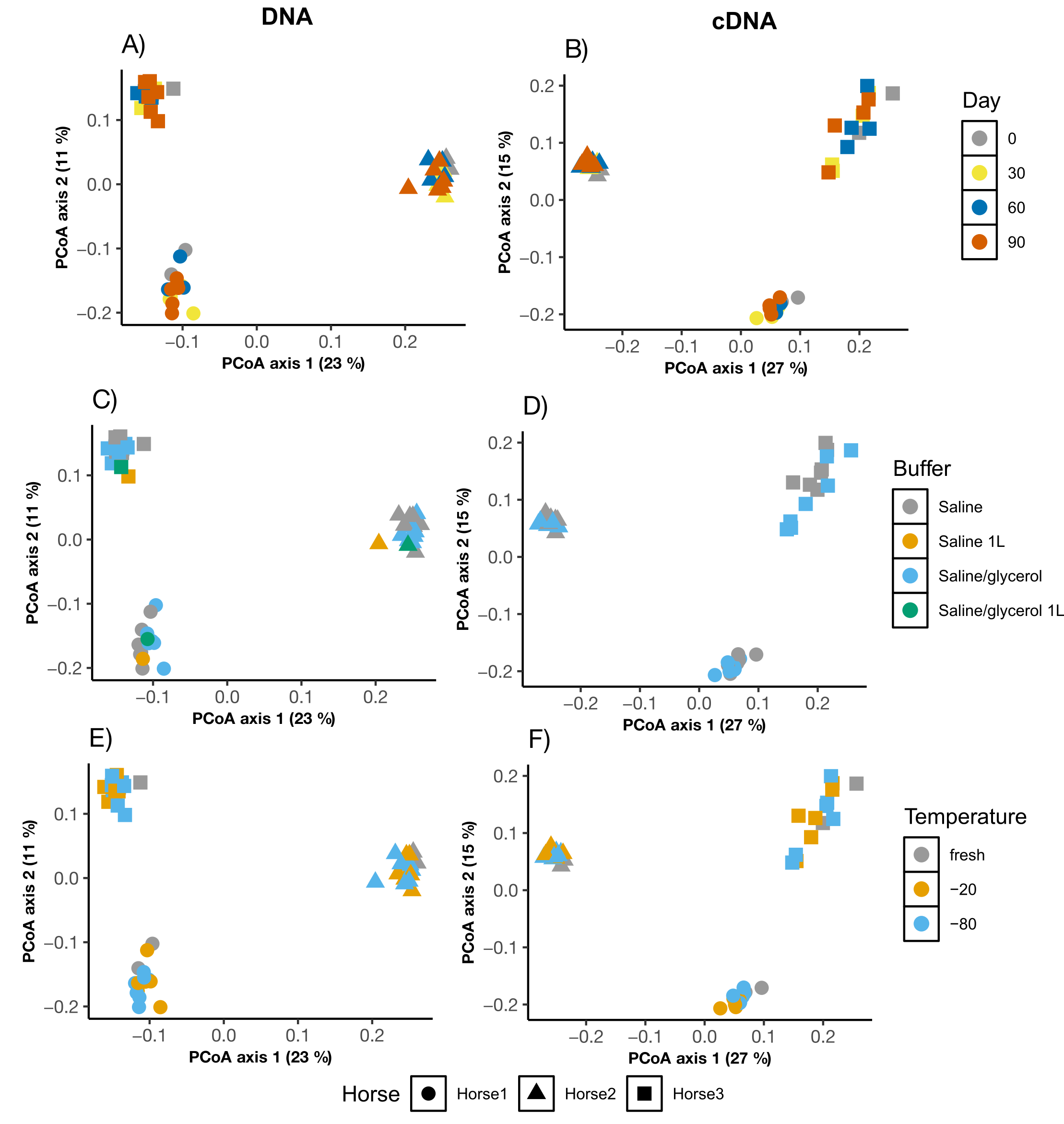

Supplement: Supplementary file 4 — Additional Fig. 3: Measurement of bacterial community composition (beta diversity) for individual storage variables Description: The principal coordinate analysis (PCoA) plot shows unweighted UniFrac distances between samples, with samples that are more similar located closer to one another. Each data point represents an individual slurry sample. For this study, there was an effect of individual horse for all variables within both DNA and cDNA analysis (A-F). Neither day of storage (A, B) nor buffer type (C, D) showed significant similarities for bacterial community. Fresh samples were more similar to each other in community composition than matched frozen samples (E, F) [file 12917_2024_4166_MOESM4_ESM.tif]

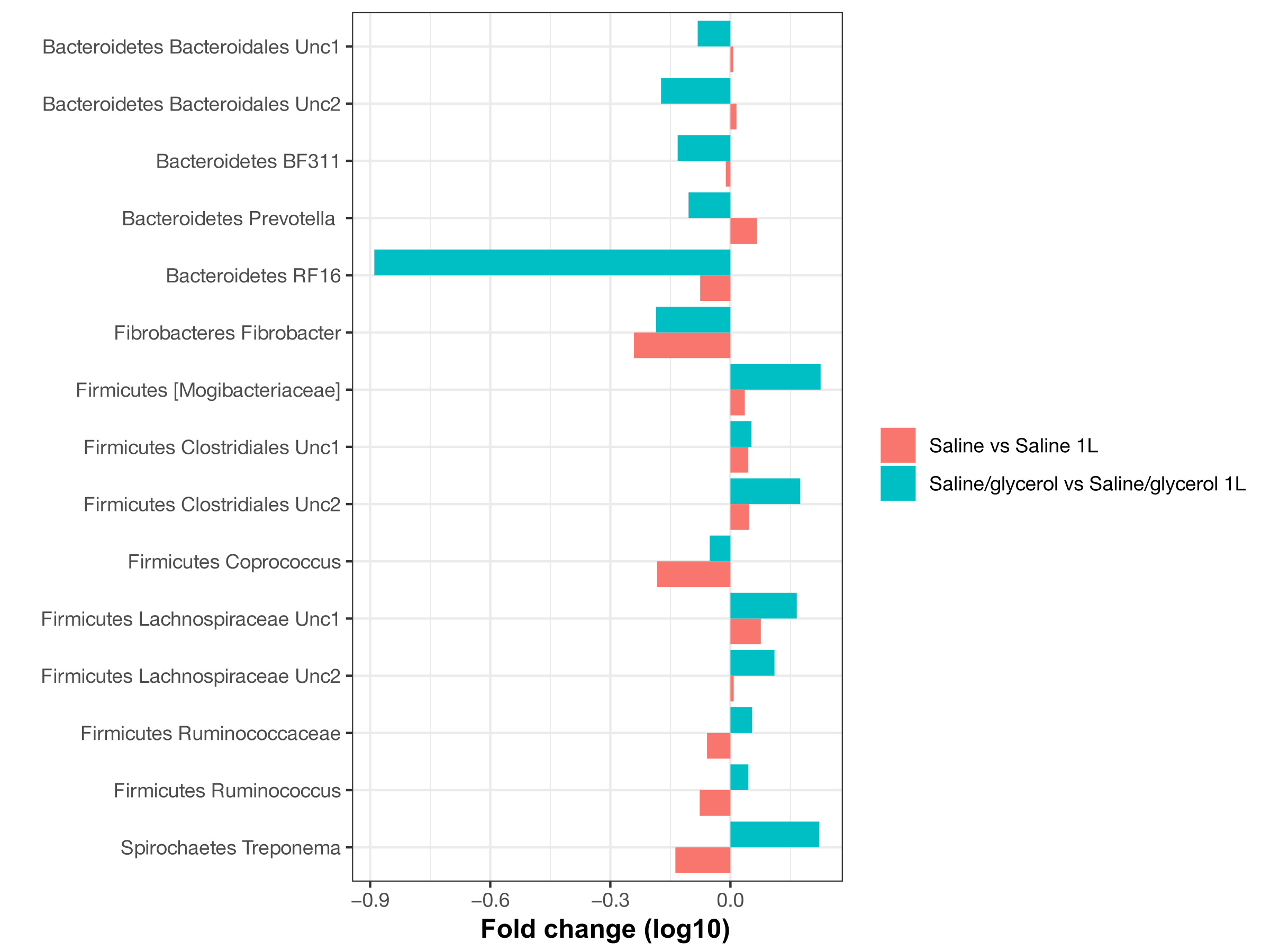

Supplement: Supplementary file 5 — Additional Fig. 4: Fold change in the relative abundance of genera common to both DNA-based and cDNA-based analysis for 1-liter storage size Description: Included genera have a mean relative abundance of > 1%. The 1-liter aliquot size was only stored at -80 °C and only underwent DNA-based analysis. The Y axis represents the fold change in relative abundance (log10). The X axis represents the individual genera, grouped by two colored bars which indicate the fold change for each buffer type (saline or saline plus glycerol) [file 12917_2024_4166_MOESM5_ESM.tif]
