# Supplementary material for: Assessment of fecal bacterial viability and diversity in fresh and frozen fecal microbiota transplant (FMT) product in horses
Source: BMC Vet Res. 2024 Jul 10;20:306. doi: 10.1186/s12917-024-04166-w (PMC11234551; doi:10.1186/s12917-024-04166-w)
Supplement: Supplementary file 6 — Additional Table 1: Effects of storage variables and their interactions on alpha diversity indices within each frozen storage temperature and extraction type [file 12917_2024_4166_MOESM6_ESM.docx]

|  | **DNA** | | | | | | | **cDNA** | | | | | |
| --- | --- | --- | --- | --- | --- | --- | --- | --- | --- | --- | --- | --- | --- |
|  | **-20°C** | | | **-80°C** | | | | **-20°C** | | | **-80°C** | | |
|  | **Species Richness** | **Shannon Diversity** | **Species Richness** | | **Shannon Diversity** | | **Species Richness** | | **Shannon Diversity** | **Species Richness** | | **Shannon Diversity** |  |
| **Buffer** | 0.605 | 0.858 | 0.654 | | 0.959 | | 0.476 | | 0.753 | 0.853 | | 0.901 |  |
| **Day** | 0.107 | 0.210 | 0.020 | | 0.022 | | 0.988 | | 0.827 | 0.944 | | 0.575 |  |
| **Buffer*Day** | 0.798 | 0.827 | 0.918 | | 0.678 | | 0.649 | | 0.637 | 0.587 | | 0.944 |  |
|  | | | | | | | | | | | | | |
| **D0 vs. D30** | | | | | | | | | | | | | |
| **Buffer** | 0.605 | 0.858 | 0.522 | | 0.876 | | 0.861 | | 0.937 | 0.804 | | 0.899 |  |
| **Day** | 0.107 | 0.210 | 0.120 | | 0.104 | | 0.880 | | 0.810 | 0.889 | | 0.326 |  |
| **Buffer*Day** | 0.798 | 0.827 | 0.803 | | 0.937 | | 0.648 | | 0.972 | 0.510 | | 0.999 |  |
|  | | | | | | | | | | | | | |
| **D0 vs. D60** | | | | | | | | | | | | | |
| **Buffer** | 0.385 | 0.676 | 0.292 | | 0.579 | | 0.996 | | 0.819 | 0.996 | | 0.819 |  |
| **Day** | 0.697 | 0.821 | 0.080 | | 0.050 | | 0.485 | | 0.256 | 0.485 | | 0.256 |  |
| **Buffer*Day** | 0.182 | 0.317 | 0.892 | | 0.808 | | 0.741 | | 0.671 | 0.741 | | 0.671 |  |
|  | | | | | | | | | | | | | |
| **D0 vs. D90** | | | | | | | | | | | | | |
| **Buffer** | 0.579 | 0.999 | 0.636 | | 0.991 | | 0.973 | | 0.891 | 0.973 | | 0.891 |  |
| **Day** | 0.095 | 0.069 | 0.035 | | 0.030 | | 0.987 | | 0.698 | 0.987 | | 0.698 |  |
| **Buffer*Day** | 0.637 | 0.318 | 0.665 | | 0.431 | | 0.958 | | 0.738 | 0.958 | | 0.738 |  |
|  | | | | | | | | | | | | | |
| **D0 vs. D90 1L** | | | | | | | | | | | | | |
| **Buffer** | Did not perform | | | 0.580 | | 0.841 | | Did not perform | | | | | |
| **Day** |  |  |  | P < 0.001 | | 0.001 | |  |  |  |  |  |  |
| **Buffer*Day** |  |  |  | 0.139 | | 0.060 | |  |  |  |  |  |  |

D0 = fresh sample, 1L = 1-liter aliquot size
