# Supplementary material for: Assessment of fecal bacterial viability and diversity in fresh and frozen fecal microbiota transplant (FMT) product in horses
Source: BMC Vet Res. 2024 Jul 10;20:306. doi: 10.1186/s12917-024-04166-w (PMC11234551; doi:10.1186/s12917-024-04166-w)
Supplement: Supplementary file 7 — Additional Table 2: Effects of individual storage variables on beta diversity patterns within each frozen storage temperature and extraction type [file 12917_2024_4166_MOESM7_ESM.docx]

|  | **DNA** | | | | **cDNA** | | | |
| --- | --- | --- | --- | --- | --- | --- | --- | --- |
|  | **-20°C** | | **-80°C** | | **-20°C** | | **-80°C** | |
|  | **WU** | **UWU** | **WU** | **UWU** | **WU** | **UWU** | **WU** | **UWU** |
| **D0 vs. D30** | | | | | | | | |
| **Buffer** | 0.062 | 0.725 | 0.036 | 0.216 | 0.423 | 0.976 | 0.807 | 0.858 |
| **Day** | 0.191 | 0.007 | 0.113 | 0.049 | 0.003 | 0.008 | 0.003 | 0.008 |
| **Buffer*Day** | 0.185 | 0.931 | 0.113 | 0.448 | 0.135 | 0.471 | 0.247 | 0.637 |
|  | | | | | | | | |
| **D0 vs. D60** | | | | | | | | |
| **Buffer** | 0.133 | 0.292 | 0.320 | 0.307 | 0.759 | 0.867 | 0.914 | 0.980 |
| **Day** | 0.001 | 0.033 | 0.010 | 0.038 | 0.003 | 0.004 | 0.007 | 0.007 |
| **Buffer*Day** | 0.518 | 0.436 | 0.370 | 0.319 | 0.317 | 0.437 | 0.497 | 0.240 |
|  | | | | | | | | |
| **D0 vs. D90** | | | | | | | | |
| **Buffer** | 0.118 | 0.058 | 0.033 | 0.285 | 0.742 | 0.393 | 0.746 | 0.694 |
| **Day** | 0.001 | 0.121 | 0.071 | 0.004 | 0.003 | 0.003 | 0.018 | 0.005 |
| **Buffer*Day** | 0.319 | 0.560 | 0.216 | 0.639 | 0.408 | 0.752 | 0.356 | 0.486 |
|  | | | | | | | | |
| **D0 vs. D90 1L** | | | | | | | | |
| **Buffer** | Did not perform | | 0.134 | 0.211 | Did not perform | | | |
| **Day** |  |  | 0.001 | 0.001 |  |  |  |  |
| **Buffer*Day** |  |  | 0.130 | 0.250 |  |  |  |  |

WU = weighted UniFrac distance, UWU = unweighted UniFrac distance, D0 = fresh sample, 1L = 1-liter aliquot size
