# Supplementary material for: Assessment of fecal bacterial viability and diversity in fresh and frozen fecal microbiota transplant (FMT) product in horses
Source: BMC Vet Res. 2024 Jul 10;20:306. doi: 10.1186/s12917-024-04166-w (PMC11234551; doi:10.1186/s12917-024-04166-w)
Supplement: Supplementary file 8 — Additional Table 3: Mean relative abundance of phyla (percentage, %) depicted by day and temperature for each buffer in DNA and cDNA [file 12917_2024_4166_MOESM8_ESM.docx]

|  | | Saline | | | | | | | | Saline plus glycerol | | | | | | | |
| --- | --- | --- | --- | --- | --- | --- | --- | --- | --- | --- | --- | --- | --- | --- | --- | --- | --- |
|  |  |  | -20 | | | -80 | | | |  | -20 | | | -80 | | | |
|  | **Phylum** | **D0** | **D30** | **D60** | **D90** | **D30** | **D60** | **D90** | **D90 1L** | **D0** | **D30** | **D60** | **D90** | **D30** | **D60** | **D90** | **D90 1L** |
| DNA | Firmicutes | 51.85 | 59.1 | 61 | 62.5 | 58.2 | 58.7 | 59.2 | 63.82 | 52.1 | 51.1 | 54.1 | 55.3 | 49.9 | 54.4 | 53.2 | 54.58 |
|  | Bacteroidetes | 38.95 | 34 | 30.5 | 31.5 | 35.5 | 34.5 | 35.2 | 28.17 | 36.9 | 39.5 | 34.9 | 35.8 | 40.2 | 36.1 | 37.2 | 37.33 |
|  | Fibrobacteres | 2.31 | 0.6 | 1.98 | 0.38 | 0.9 | 1.59 | 0.89 | 1.51 | 3.83 | 3.02 | 3.83 | 1.95 | 3.21 | 2.39 | 3.03 | 2.2 |
|  | Tenericutes | 1.58 | 1.31 | 1.09 | 1.26 | 0.85 | 0.66 | 0.7 | 0.89 | 1.54 | 1.47 | 1.16 | 1.59 | 1.68 | 1.1 | 1.68 | 1.34 |
|  | Spirochaetes | 1.38 | 1.13 | 2 | 1.19 | 1.18 | 1.98 | 0.92 | 1.79 | 1.94 | 1.62 | 3.36 | 1.92 | 1.5 | 2.97 | 1.67 | 1.43 |
|  | Proteobacteria | 1.3 | 1.18 | 0.88 | 0.87 | 1.16 | 0.7 | 0.96 | 0.72 | 1.27 | 1.2 | 0.82 | 1.26 | 1.22 | 0.86 | 1.2 | 0.99 |
|  | Actinobacteria | 0.85 | 0.96 | 1.24 | 0.93 | 1.01 | 1.21 | 1.13 | 1.87 | 0.76 | 0.59 | 0.89 | 0.74 | 0.78 | 0.85 | 0.75 | 0.93 |
|  |  |  |  |  |  |  |  |  |  |  |  |  |  |  |  |  |  |
|  |  | **D0** | **D30** | **D60** | **D90** | **D30** | **D60** | **D90** | **D90 1L** | **D0** | **D30** | **D60** | **D90** | **D30** | **D60** | **D90** | **D90 1L** |
| cDNA | Firmicutes | 70.42 | 75.2 | 75.3 | 76.4 | 74.2 | 74.2 | 74.9 | DNP | 71.4 | 64.8 | 71.8 | 72.5 | 68.6 | 71 | 70.8 | DNP |
|  | Bacteroidetes | 18.03 | 15.8 | 16.9 | 16.1 | 17.8 | 17.7 | 17.3 |  | 17.1 | 22 | 18.2 | 18 | 19.8 | 18.2 | 18.6 |  |
|  | Fibrobacteres | 4.78 | 2.95 | 1.68 | 1.46 | 2.56 | 2.54 | 2.47 |  | 4.52 | 6.75 | 4.1 | 3.69 | 4.95 | 4.53 | 4.52 |  |
|  | Tenericutes | 0.27 | 0.39 | 0.42 | 0.36 | 0.27 | 0.32 | 0.26 |  | 0.32 | 0.46 | 0.42 | 0.4 | 0.36 | 0.34 | 0.37 |  |
|  | Spirochaetes | 4.4 | 2.44 | 2.54 | 2.77 | 2.23 | 2.14 | 2.3 |  | 4.42 | 2.76 | 2.63 | 2.22 | 2.92 | 3 | 2.79 |  |
|  | Proteobacteria | 0.81 | 1.05 | 0.93 | 0.84 | 0.99 | 1.06 | 1 |  | 0.89 | 1.26 | 1 | 1.15 | 1.18 | 1.05 | 1.05 |  |
|  | Actinobacteria | 0.36 | 0.63 | 0.67 | 0.72 | 0.53 | 0.6 | 0.52 |  | 0.37 | 0.35 | 0.47 | 0.36 | 0.42 | 0.48 | 0.46 |  |

Phyla with >1% mean relative abundance at any storage time or condition

DNP = did not perform
