# Supplementary material for: Assessment of fecal bacterial viability and diversity in fresh and frozen fecal microbiota transplant (FMT) product in horses
Source: BMC Vet Res. 2024 Jul 10;20:306. doi: 10.1186/s12917-024-04166-w (PMC11234551; doi:10.1186/s12917-024-04166-w)
Supplement: Supplementary file 9 — Additional Table 4a: Mean relative abundance (percentage, %) of common genera between DNA-based and cDNA-based analysis, DNA-analysis [file 12917_2024_4166_MOESM9_ESM.docx]

|  | **Saline** | | | | | | | | **Saline Plus Glycerol** | | | | | | | |
| --- | --- | --- | --- | --- | --- | --- | --- | --- | --- | --- | --- | --- | --- | --- | --- | --- |
|  | **Fresh** | **-20°C** | | | **-80°C** | | | | **Fresh** | **-20°C** | | | **-80°C** | | | |
| **Phylum**  *Genus* | **D0** | **D30** | **D60** | **D90** | **D30** | **D60** | **D90** | **D90 1L** | **D0** | **D30** | **D60** | **D90** | **D30** | **D60** | **D90** | **D90 1L** |
| **Bacteria (unclassified)** | 0.39 | 0.15 | 0.18 | 0.19 | 0.19 | 0.06 | 0.16 | 0.11 | 0.36 | 0.25 | 0.23 | 0.27 | 0.34 | 0.25 | 0.35 | 0.26 |
| **Actinobacteria** |  | | | | | | | | | | | | | | | |
| *Adlercreutzia* | 0.22 | 0.32 | 0.47 | 0.28 | 0.34 | 0.32 | 0.34 | 0.54 | 0.23 | 0.14 | 0.27 | 0.21 | 0.18 | 0.31 | 0.16 | 0.27 |
| Coriobacteriaceae 1 (unclassified) | 0.12 | 0.13 | 0.2 | 0.16 | 0.16 | 0.25 | 0.22 | 0.26 | 0.1 | 0.05 | 0.18 | 0.14 | 0.14 | 0.18 | 0.14 | 0.19 |
| Coriobacteriaceae 1 (unclassified) | 0.4 | 0.43 | 0.48 | 0.4 | 0.47 | 0.56 | 0.51 | 0.88 | 0.38 | 0.33 | 0.4 | 0.33 | 0.38 | 0.33 | 0.41 | 0.38 |
| **Armatimonadetes** |  | | | | | | | | | | | | | | | |
| RB046 (unclassified) | 0.04 | 0.07 | 0.06 | 0.06 | 0.07 | 0.05 | 0.03 | 0.06 | 0.03 | 0.08 | 0.03 | 0.04 | 0.04 | 0.05 | 0.04 | 0.06 |
| **Bacteroidetes** |  | | | | | | | | | | | | | | | |
| Bacteroidales 1 (unclassified) | 2.84 | 2.48 | 1.88 | 1.98 | 2.8 | 2.59 | 2.97 | 1.9 | 2.78 | 3.13 | 2.76 | 2.79 | 2.95 | 2.89 | 3.08 | 2.88 |
| Bacteroidales 2 (unclassified) | 19.16 | 18.31 | 16.48 | 17.09 | 18.42 | 18.65 | 18.66 | 15.87 | 18.62 | 19.5 | 18.2 | 18.53 | 20.58 | 19.98 | 18.16 | 18.91 |
| *Bacteroides* | 0.05 | 0.07 | 0.05 | 0.08 | 0.06 | 0.08 | 0.09 | 0.04 | 0.09 | 0.1 | 0.11 | 0.04 | 0.1 | 0.1 | 0.06 | 0.07 |
| *BF311* | 1.94 | 2.16 | 1.65 | 2.01 | 1.74 | 1.34 | 1.95 | 1.43 | 1.89 | 2.43 | 1.42 | 2.02 | 2.01 | 1.2 | 1.81 | 1.84 |
| *CF231* | 1.58 | 1.79 | 1.67 | 1.6 | 2.11 | 1.88 | 1.64 | 1.66 | 1.59 | 1.79 | 1.49 | 1.54 | 1.89 | 0.82 | 1.7 | 1.55 |
| Marinilabiaceae (unclassified) | 0.21 | 0.13 | 0.12 | 0.11 | 0.15 | 0.22 | 0.12 | 0.12 | 0.25 | 0.2 | 0.24 | 0.18 | 0.28 | 0.27 | 0.21 | 0.2 |
| *Paludibacter* | 1.8 | 0.89 | 1.05 | 0.91 | 1.16 | 0.79 | 0.73 | 0.56 | 1.59 | 1.93 | 1.19 | 1.24 | 1.66 | 0.54 | 1.76 | 1.36 |
| Paraprevotellaceae 1 (unclassified) | 0.29 | 0.28 | 0.22 | 0.26 | 0.22 | 0.3 | 0.26 | 0.14 | 0.23 | 0.24 | 0.27 | 0.19 | 0.29 | 0.18 | 0.26 | 0.24 |
| Paraprevotellaceae 2 (unclassified) | 0.57 | 0.57 | 0.37 | 0.56 | 0.6 | 0.56 | 0.55 | 0.33 | 0.54 | 0.7 | 0.51 | 0.53 | 0.64 | 0.36 | 0.63 | 0.69 |
| *Prevotella 1* | 0.17 | 0.16 | 0.16 | 0.11 | 0.16 | 0.24 | 0.23 | 0.16 | 0.23 | 0.15 | 0.17 | 0.14 | 0.13 | 0.12 | 0.23 | 0.2 |
| *Prevotella 2* | 2.96 | 2.72 | 2.21 | 2.32 | 3.06 | 3.07 | 3.01 | 2.32 | 2.79 | 3.02 | 2.82 | 2.6 | 2.86 | 3.7 | 3.02 | 3.25 |
| RF16 (unclassified) | 3.93 | 1.52 | 1.44 | 1.42 | 1.37 | 1.15 | 1.28 | 0.51 | 3.44 | 3.3 | 2.49 | 2.88 | 3.3 | 2.39 | 2.96 | 2.89 |
| S24-7 (unclassified) | 0.21 | 0.37 | 0.35 | 0.27 | 0.24 | 0.24 | 0.29 | 0.33 | 0.25 | 0.24 | 0.2 | 0.18 | 0.24 | 0.22 | 0.18 | 0.21 |
| *YRC22* | 1.93 | 1.83 | 1.85 | 2.04 | 2.51 | 2.81 | 2.65 | 2.09 | 1.71 | 1.73 | 2.09 | 1.89 | 1.96 | 2.03 | 1.93 | 2.05 |
| **Cyanobacteria** |  | | | | | | | | | | | | | | | |
| YS2 (unclassified) | 0.82 | 0.9 | 0.58 | 0.66 | 0.57 | 0.31 | 0.34 | 0.2 | 0.77 | 0.76 | 0.41 | 0.68 | 0.67 | 0.65 | 0.43 | 0.44 |
| **Fibrobacteres** |  | | | | | | | | | | | | | | | |
| *Fibrobacter* | 2.31 | 0.6 | 1.98 | 0.38 | 0.9 | 1.59 | 0.89 | 1.51 | 3.83 | 3.02 | 3.83 | 1.95 | 3.21 | 2.39 | 3.03 | 2.2 |
| **Firmicutes** |  | | | | | | | | | | | | | | | |
| *Anaerovibrio* | 0.07 | 0.02 | 0.04 | 0.03 | 0.04 | 0.06 | 0.1 | 0.01 | 0.06 | 0.04 | 0.02 | 0.05 | 0.03 | 0.02 | 0.08 | 0.03 |
| *Bulleidia* | 0.13 | 0.13 | 0.17 | 0.17 | 0.14 | 0.29 | 0.24 | 0.34 | 0.16 | 0.15 | 0.23 | 0.2 | 0.15 | 0.18 | 0.14 | 0.27 |
| Christensenellaceae (unclassified) | 0.34 | 0.38 | 0.52 | 0.32 | 0.38 | 0.53 | 0.27 | 0.52 | 0.35 | 0.26 | 0.42 | 0.35 | 0.42 | 0.34 | 0.31 | 0.33 |
| Clostridiales 1 (unclassified) | 9.46 | 10.37 | 10.58 | 12.33 | 10.18 | 10.11 | 10.85 | 10.68 | 8.46 | 8.54 | 8.67 | 9.74 | 8.42 | 9.62 | 8.87 | 9.38 |
| Clostridiales 2 (unclassified) | 4.59 | 5.88 | 5.72 | 5.68 | 5.72 | 5.44 | 4.93 | 6.86 | 4.71 | 4.61 | 4.86 | 4.84 | 4.36 | 4.68 | 4.88 | 5.24 |
| *Clostridium* | 0.41 | 0.48 | 0.58 | 0.42 | 0.5 | 0.56 | 0.59 | 0.57 | 0.3 | 0.32 | 0.38 | 0.35 | 0.33 | 0.43 | 0.28 | 0.39 |
| *Coprococcus* | 1.01 | 0.98 | 1.6 | 1.35 | 0.9 | 0.86 | 0.97 | 0.9 | 1.3 | 0.72 | 1.23 | 1.03 | 1.07 | 0.94 | 1.22 | 0.85 |
| *Dorea* | 0.1 | 0.06 | 0.17 | 0.12 | 0.06 | 0.14 | 0.05 | 0.11 | 0.08 | 0.09 | 0.19 | 0.09 | 0.06 | 0.15 | 0.07 | 0.06 |
| *Epulopiscium* | 0.18 | 0.05 | 0.12 | 0.14 | 0.12 | 0.17 | 0.13 | 0.11 | 0.13 | 0.16 | 0.14 | 0.11 | 0.11 | 0.1 | 0.19 | 0.07 |
| Erysipelotrichaceae 1 (unclassified) | 0.07 | 0.06 | 0.06 | 0.05 | 0.1 | 0.05 | 0.05 | 0.02 | 0.05 | 0.05 | 0.05 | 0.07 | 0.05 | 0.07 | 0.05 | 0.05 |
| Erysipelotrichaceae 2 (unclassified) | 0.62 | 0.48 | 0.6 | 0.52 | 0.55 | 0.61 | 0.58 | 0.5 | 0.73 | 0.59 | 0.67 | 0.68 | 0.68 | 0.71 | 0.67 | 0.65 |
| *Eubacterium* | 0.95 | 1.13 | 1.78 | 1.55 | 1.02 | 1.88 | 1.85 | 1.55 | 0.97 | 1.07 | 1.69 | 1.07 | 0.9 | 1.34 | 1.01 | 1.26 |
| Firmicutes (unclassified) | 0.03 | 0.02 | 0.03 | 0.05 | 0.02 | 0.02 | 0.01 | 0.02 | 0.05 | 0.02 | 0.02 | 0.03 | 0.04 | 0.03 | 0.05 | 0.03 |
| Lachnospiraceae 1 (unclassified) | 8.09 | 9.73 | 9.39 | 11.39 | 9.43 | 8.42 | 9.92 | 10.42 | 7.62 | 7.39 | 7.09 | 8.21 | 7.07 | 8.23 | 8.44 | 7.77 |
| Lachnospiraceae 2 (unclassified) | 7.35 | 9.07 | 10.54 | 9.57 | 9.74 | 10.31 | 10.49 | 10.76 | 6.91 | 7.43 | 9.39 | 8 | 6.41 | 9.14 | 7.69 | 8.23 |
| *Lactobacillus* | 0.23 | 0.37 | 0.55 | 0.48 | 0.49 | 0.81 | 0.52 | 1.05 | 0.32 | 0.34 | 0.69 | 0.45 | 0.4 | 0.46 | 0.19 | 0.7 |
| Mogibacteriaceae (unclassified) | 2.01 | 2.34 | 3.05 | 2.84 | 2.68 | 2.88 | 2.46 | 3.37 | 2.17 | 2.08 | 2.56 | 2.4 | 2.01 | 2.11 | 2.2 | 2.35 |
| *Oscillospira* | 0.62 | 0.68 | 0.6 | 0.48 | 0.61 | 0.67 | 0.52 | 1.02 | 0.62 | 0.7 | 0.45 | 0.51 | 0.57 | 0.35 | 0.66 | 0.47 |
| *p-75-a5* | 0.4 | 0.48 | 0.46 | 0.5 | 0.37 | 0.38 | 0.46 | 0.64 | 0.37 | 0.53 | 0.28 | 0.39 | 0.4 | 0.45 | 0.49 | 0.4 |
| *Phascolarctobacterium* | 1.73 | 1.93 | 1.33 | 1.72 | 2.24 | 2.63 | 2.2 | 1.73 | 1.8 | 2.16 | 2.17 | 1.86 | 2.45 | 2.25 | 1.77 | 2.45 |
| *Pseudoramibacter Eubacterium* | 0.14 | 0.13 | 0.12 | 0.13 | 0.1 | 0.08 | 0.13 | 0.23 | 0.09 | 0.04 | 0.07 | 0.06 | 0.12 | 0.07 | 0.06 | 0.09 |
| *RFN20* | 2.7 | 1.7 | 1.55 | 1.49 | 1.16 | 0.74 | 0.84 | 0.3 | 2.33 | 2.35 | 1.83 | 2.51 | 2.47 | 1.66 | 2.35 | 2.22 |
| *Roseburia* | 0.1 | 0.05 | 0.11 | 0.07 | 0.19 | 0.24 | 0.13 | 0 | 0.13 | 0.07 | 0.08 | 0.06 | 0.07 | 0.14 | 0.11 | 0.19 |
| Ruminococcaceae 1 (unclassified) | 0.83 | 1.1 | 0.96 | 0.87 | 0.93 | 0.89 | 0.96 | 1.18 | 0.96 | 0.92 | 0.84 | 1.01 | 0.94 | 0.87 | 0.99 | 0.91 |
| Ruminococcaceae 2 (unclassified) | 6.91 | 8.23 | 7.93 | 7.48 | 7.21 | 7.06 | 7.71 | 7.82 | 8.33 | 7.71 | 7.49 | 8.25 | 7.3 | 7.52 | 7.33 | 7.28 |
| *Ruminococcus* | 1.71 | 1.99 | 1.59 | 1.62 | 2.13 | 1.84 | 1.05 | 1.9 | 2.06 | 1.7 | 1.67 | 1.71 | 2.2 | 1.52 | 1.91 | 1.72 |
| *Streptococcus* | 0 | 0 | 0.02 | 0.02 | 0.02 | 0.01 | 0.02 | 0.01 | 0 | 0.01 | 0.01 | 0.01 | 0.01 | 0.02 | 0.01 | 0.02 |
| Veillonellaceae (unclassified) | 0.82 | 1.04 | 0.49 | 0.79 | 0.91 | 0.67 | 1.01 | 0.96 | 0.79 | 0.83 | 0.65 | 0.98 | 0.66 | 0.76 | 0.98 | 0.86 |
| **Proteobacteria** |  | | | | | | | | | | | | | | | |
| Alphaproteobacteria (unclassified) | 0.69 | 0.58 | 0.38 | 0.46 | 0.53 | 0.3 | 0.38 | 0.3 | 0.56 | 0.57 | 0.32 | 0.58 | 0.58 | 0.4 | 0.54 | 0.46 |
| GMD14H09 (unclassified) | 0.27 | 0.32 | 0.25 | 0.18 | 0.36 | 0.24 | 0.26 | 0.32 | 0.36 | 0.31 | 0.28 | 0.33 | 0.38 | 0.18 | 0.34 | 0.3 |
| Rickettsiales (unclassified) | 0.06 | 0.06 | 0.05 | 0.04 | 0.04 | 0.02 | 0.07 | 0.01 | 0.04 | 0.05 | 0.05 | 0.05 | 0.04 | 0.04 | 0.06 | 0.03 |
| *Sutterella* | 0.03 | 0.04 | 0.04 | 0.02 | 0.04 | 0.02 | 0.06 | 0.01 | 0.07 | 0.05 | 0.03 | 0.06 | 0.06 | 0.02 | 0.01 | 0.05 |
| **Spirochaetes** |  | | | | | | | | | | | | | | | |
| *Sphaerochaeta* | 0.26 | 0.22 | 0.17 | 0.26 | 0.14 | 0.12 | 0.11 | 0.1 | 0.26 | 0.17 | 0.19 | 0.26 | 0.25 | 0.19 | 0.23 | 0.23 |
| *Treponema* | 1 | 0.83 | 1.7 | 0.82 | 0.96 | 1.82 | 0.77 | 1.66 | 1.54 | 1.39 | 3.12 | 1.54 | 1.16 | 2.73 | 1.36 | 1.12 |
| **Synergistetes** |  | | | | | | | | | | | | | | | |
| Synergistales (unclassified) | 0.22 | 0.25 | 0.26 | 0.25 | 0.22 | 0.17 | 0.24 | 0.48 | 0.15 | 0.19 | 0.16 | 0.2 | 0.16 | 0.16 | 0.22 | 0.22 |
| **Tenericutes** |  | | | | | | | | | | | | | | | |
| *Anaeroplasma* | 0.44 | 0.38 | 0.28 | 0.41 | 0.18 | 0.1 | 0.14 | 0.05 | 0.53 | 0.55 | 0.38 | 0.61 | 0.47 | 0.39 | 0.51 | 0.29 |
| Anaeroplasmataceae (unclassified) | 0.16 | 0.15 | 0.12 | 0.18 | 0.09 | 0.04 | 0.07 | 0.05 | 0.18 | 0.19 | 0.14 | 0.19 | 0.21 | 0.11 | 0.18 | 0.1 |
| Mollicutes (unclassified) | 0.06 | 0.06 | 0.07 | 0.04 | 0.05 | 0.02 | 0.02 | 0.02 | 0.09 | 0.14 | 0.07 | 0.1 | 0.1 | 0.02 | 0.14 | 0.06 |
| Mycoplasmataceae (unclassified) | 0.33 | 0.13 | 0.13 | 0.13 | 0.11 | 0.07 | 0.13 | 0.06 | 0.14 | 0.1 | 0.12 | 0.12 | 0.2 | 0.09 | 0.25 | 0.2 |
| RF39 (unclassified) | 0.49 | 0.5 | 0.44 | 0.39 | 0.37 | 0.38 | 0.27 | 0.68 | 0.51 | 0.42 | 0.37 | 0.49 | 0.59 | 0.36 | 0.52 | 0.57 |
| **Verrucomicrobia** |  | | | | | | | | | | | | | | | |
| RFP12 (unclassified) | 0.06 | 0.04 | 0.02 | 0.01 | 0 | 0.02 | 0 | 0.06 | 0.12 | 0.05 | 0.04 | 0.01 | 0.07 | 0.03 | 0.03 | 0.03 |

Genera with >75% presence across all samples
