# Supplementary material for: Assessment of fecal bacterial viability and diversity in fresh and frozen fecal microbiota transplant (FMT) product in horses
Source: BMC Vet Res. 2024 Jul 10;20:306. doi: 10.1186/s12917-024-04166-w (PMC11234551; doi:10.1186/s12917-024-04166-w)
Supplement: Supplementary file 10 — Additional Table 4b: Mean relative abundance (percentage, %) of common genera between DNA-based and cDNA-based analysis, cDNA-analysis [file 12917_2024_4166_MOESM10_ESM.docx]

|  | **Saline** | | | | | | | **Saline Plus Glycerol** | | | | | | |
| --- | --- | --- | --- | --- | --- | --- | --- | --- | --- | --- | --- | --- | --- | --- |
|  | **Fresh** | **-20°C** | | | **-80°C** | | | **Fresh** | **-20°C** | | | **-80°C** | | |
| **Phylum**  *Genus* | **D0** | **D30** | **D60** | **D90** | **D30** | **D60** | **D90** | **D0** | **D30** | **D60** | **D90** | **D30** | **D60** | **D90** |
| **Bacteria (unclassified)** | 0.26 | 0.26 | 0.27 | 0.23 | 0.19 | 0.23 | 0.19 | 0.26 | 0.38 | 0.32 | 0.38 | 0.35 | 0.32 | 0.32 |
| **Actinobacteria** |  |  |  |  |  |  |  |  |  |  |  |  |  |  |
| *Adlercreutzia* | 0.03 | 0.06 | 0.06 | 0.07 | 0.05 | 0.07 | 0.05 | 0.06 | 0.03 | 0.04 | 0.02 | 0.02 | 0.01 | 0.04 |
| Coriobacteriaceae 1 (unclassified) | 0 | 0.01 | 0.03 | 0.01 | 0.02 | 0.01 | 0 | 0.01 | 0.01 | 0.01 | 0.01 | 0.02 | 0.03 | 0.01 |
| Coriobacteriaceae 1 (unclassified) | 0.27 | 0.48 | 0.53 | 0.53 | 0.39 | 0.44 | 0.4 | 0.26 | 0.25 | 0.37 | 0.28 | 0.34 | 0.35 | 0.34 |
| **Armatimonadetes** |  |  |  |  |  |  |  |  |  |  |  |  |  |  |
| RB046 (unclassified) | 0.02 | 0.06 | 0.05 | 0.07 | 0.04 | 0.04 | 0.03 | 0.01 | 0.05 | 0.03 | 0.04 | 0.04 | 0.02 | 0.03 |
| **Bacteroidetes** |  |  |  |  |  |  |  |  |  |  |  |  |  |  |
| Bacteroidales 1 (unclassified) | 1.91 | 1.32 | 1.44 | 1.39 | 1.69 | 1.63 | 1.48 | 1.77 | 1.95 | 1.54 | 1.65 | 1.8 | 1.68 | 1.76 |
| Bacteroidales 2 (unclassified) | 8.51 | 7.15 | 7.67 | 7.38 | 7.9 | 8.13 | 8.09 | 8.1 | 9.79 | 8.17 | 7.79 | 8.66 | 7.71 | 8.42 |
| *Bacteroides* | 0.01 | 0.01 | 0.01 | 0 | 0.02 | 0.02 | 0.02 | 0.02 | 0.01 | 0.03 | 0.01 | 0.02 | 0.03 | 0.02 |
| *BF311* | 1.08 | 1.28 | 1.33 | 1.13 | 1.43 | 1.36 | 1.23 | 1.07 | 1.89 | 1.22 | 1.58 | 1.62 | 1.57 | 1.36 |
| *CF231* | 0.62 | 0.63 | 0.62 | 0.59 | 0.92 | 0.86 | 0.67 | 0.49 | 0.9 | 0.85 | 0.85 | 0.88 | 0.89 | 0.81 |
| Marinilabiaceae (unclassified) | 0.15 | 0.07 | 0.07 | 0.06 | 0.08 | 0.05 | 0.08 | 0.1 | 0.17 | 0.06 | 0.08 | 0.14 | 0.1 | 0.09 |
| Paludibacter | 0.51 | 0.74 | 0.68 | 0.64 | 0.64 | 0.75 | 0.76 | 0.65 | 1.14 | 0.76 | 0.68 | 0.77 | 0.63 | 0.79 |
| Paraprevotellaceae 1 (unclassified) | 0.15 | 0.17 | 0.12 | 0.12 | 0.19 | 0.21 | 0.15 | 0.14 | 0.2 | 0.21 | 0.18 | 0.19 | 0.2 | 0.19 |
| Paraprevotellaceae 2 (unclassified) | 0.29 | 0.17 | 0.21 | 0.19 | 0.26 | 0.27 | 0.22 | 0.26 | 0.31 | 0.28 | 0.31 | 0.31 | 0.25 | 0.28 |
| *Prevotella 1* | 0.18 | 0.09 | 0.12 | 0.13 | 0.19 | 0.14 | 0.12 | 0.12 | 0.18 | 0.15 | 0.17 | 0.15 | 0.17 | 0.14 |
| *Prevotella 2* | 2.15 | 1.57 | 1.81 | 1.73 | 1.89 | 1.81 | 1.92 | 2.04 | 2.15 | 2.11 | 1.88 | 2.13 | 2.17 | 1.83 |
| RF16 (unclassified) | 1.04 | 1.38 | 1.36 | 1.19 | 1.37 | 1.39 | 1.36 | 1.09 | 1.75 | 1.57 | 1.4 | 1.68 | 1.41 | 1.61 |
| S24-7 (unclassified) | 0.07 | 0.07 | 0.1 | 0.08 | 0.06 | 0.05 | 0.05 | 0.03 | 0.08 | 0.07 | 0.09 | 0.08 | 0.06 | 0.08 |
| *YRC22* | 0.96 | 0.78 | 0.95 | 0.99 | 0.87 | 0.73 | 0.83 | 0.89 | 1.01 | 0.79 | 0.87 | 0.9 | 0.98 | 0.77 |
| **Cyanobacteria** |  |  |  |  |  |  |  |  |  |  |  |  |  |  |
| YS2 (unclassified) | 0.34 | 0.67 | 0.73 | 0.64 | 0.64 | 0.59 | 0.58 | 0.31 | 0.68 | 0.43 | 0.8 | 0.54 | 0.46 | 0.51 |
| **Fibrobacteres** |  |  |  |  |  |  |  |  |  |  |  |  |  |  |
| *Fibrobacter* | 4.78 | 2.95 | 1.68 | 1.46 | 2.56 | 2.54 | 2.47 | 4.52 | 6.75 | 4.1 | 3.69 | 4.95 | 4.53 | 4.52 |
| **Firmicutes** |  |  |  |  |  |  |  |  |  |  |  |  |  |  |
| *Anaerovibrio* | 0.07 | 0.06 | 0.07 | 0.04 | 0.09 | 0.06 | 0.08 | 0.02 | 0.1 | 0.06 | 0.04 | 0.06 | 0.07 | 0.03 |
| *Bulleidia* | 0.13 | 0.1 | 0.08 | 0.11 | 0.12 | 0.1 | 0.12 | 0.1 | 0.09 | 0.12 | 0.09 | 0.07 | 0.06 | 0.1 |
| Christensenellaceae (unclassified) | 0.59 | 0.61 | 0.63 | 0.61 | 0.67 | 0.74 | 0.66 | 0.57 | 0.58 | 0.7 | 0.67 | 0.67 | 0.63 | 0.69 |
| Clostridiales 1 (unclassified) | 11.41 | 12.95 | 12.3 | 12.19 | 12.83 | 12.77 | 13.28 | 12.45 | 10.02 | 11.57 | 12.4 | 10.09 | 11.4 | 10.68 |
| Clostridiales 2 (unclassified) | 4.6 | 4.5 | 4.93 | 5.47 | 4.5 | 4.58 | 4.69 | 4.35 | 4.64 | 5.15 | 5.1 | 4.75 | 4.4 | 5.18 |
| *Clostridium* | 0.68 | 1.57 | 2.09 | 2.46 | 1.24 | 1.39 | 1.12 | 0.91 | 0.81 | 0.85 | 0.82 | 0.74 | 0.85 | 0.69 |
| *Coprococcus* | 2.46 | 2.49 | 2.49 | 2.41 | 2.25 | 2.24 | 2.56 | 2.74 | 1.76 | 2.11 | 2.21 | 1.93 | 2.18 | 2.12 |
| *Dorea* | 0.19 | 0.14 | 0.18 | 0.07 | 0.16 | 0.15 | 0.19 | 0.2 | 0.1 | 0.11 | 0.14 | 0.1 | 0.16 | 0.23 |
| *Epulopiscium* | 0.67 | 1.14 | 1.66 | 1.8 | 0.78 | 1.02 | 0.89 | 0.89 | 0.53 | 0.76 | 0.82 | 0.56 | 0.71 | 0.53 |
| Erysipelotrichaceae 1 (unclassified) | 0.03 | 0.04 | 0.04 | 0.04 | 0.04 | 0.03 | 0.02 | 0.02 | 0.03 | 0.02 | 0.04 | 0.03 | 0.03 | 0.02 |
| Erysipelotrichaceae 2 (unclassified) | 0.23 | 0.26 | 0.26 | 0.26 | 0.24 | 0.24 | 0.22 | 0.19 | 0.26 | 0.3 | 0.25 | 0.31 | 0.28 | 0.31 |
| *Eubacterium* | 1.56 | 1.02 | 1.06 | 1.01 | 1.17 | 1.03 | 1.09 | 1.44 | 1.03 | 1.16 | 1.11 | 1 | 0.99 | 1.32 |
| Firmicutes (unclassified) | 0.02 | 0.02 | 0.02 | 0.01 | 0.01 | 0.01 | 0 | 0.01 | 0.02 | 0.02 | 0.02 | 0.01 | 0.01 | 0.03 |
| Lachnospiraceae 1 (unclassified) | 10.7 | 12.52 | 12.09 | 11.8 | 11.43 | 11.94 | 11.82 | 11.02 | 8.61 | 9.99 | 12.34 | 8.58 | 10.01 | 9.45 |
| Lachnospiraceae 2 (unclassified) | 14.19 | 10.91 | 9.79 | 8.86 | 11.35 | 10.59 | 11.73 | 14.57 | 7.85 | 9.63 | 11.21 | 9.21 | 11.05 | 11.23 |
| *Lactobacillus* | 0.66 | 0.46 | 0.65 | 0.66 | 0.52 | 0.55 | 0.61 | 0.72 | 0.54 | 0.62 | 0.65 | 0.54 | 0.54 | 0.59 |
| Mogibacteriaceae (unclassified) | 2.87 | 3.14 | 3.73 | 3.94 | 2.99 | 2.92 | 2.91 | 3.17 | 2.63 | 3.03 | 3.12 | 2.9 | 3.14 | 3.09 |
| *Oscillospira* | 0.85 | 1.52 | 1.36 | 1.59 | 1.2 | 0.97 | 1.18 | 0.84 | 1.13 | 1.17 | 0.93 | 1.31 | 1.09 | 1.21 |
| *p-75-a5* | 0.1 | 0.18 | 0.17 | 0.24 | 0.15 | 0.21 | 0.21 | 0.11 | 0.19 | 0.16 | 0.18 | 0.18 | 0.19 | 0.17 |
| *Phascolarctobacterium* | 1.13 | 0.83 | 0.76 | 0.79 | 1.22 | 0.97 | 1.04 | 0.99 | 0.65 | 0.84 | 0.71 | 0.95 | 1.1 | 1.06 |
| *Pseudoramibacter Eubacterium* | 0.43 | 1.15 | 1.3 | 1.32 | 0.73 | 0.87 | 0.77 | 0.43 | 0.49 | 0.65 | 0.56 | 0.55 | 0.51 | 0.48 |
| *RFN20* | 0.44 | 0.68 | 0.68 | 0.67 | 0.4 | 0.5 | 0.45 | 0.5 | 0.65 | 0.77 | 0.77 | 0.67 | 0.67 | 0.75 |
| *Roseburia* | 0.24 | 0.26 | 0.25 | 0.26 | 0.24 | 0.35 | 0.24 | 0.28 | 0.21 | 0.26 | 0.29 | 0.17 | 0.16 | 0.17 |
| Ruminococcaceae 1 (unclassified) | 1.45 | 1.82 | 1.64 | 1.93 | 2 | 1.97 | 2.06 | 1.42 | 1.73 | 1.92 | 1.52 | 1.92 | 1.76 | 1.82 |
| Ruminococcaceae 2 (unclassified) | 9.81 | 11.9 | 12.31 | 13.04 | 12.38 | 12 | 11.75 | 8.76 | 14.37 | 14.29 | 11.28 | 15.28 | 13.16 | 13.07 |
| *Ruminococcus* | 4.22 | 3.89 | 3.62 | 3.72 | 4.66 | 5.11 | 4.21 | 3.94 | 4.81 | 4.71 | 4.24 | 5.34 | 5.09 | 4.92 |
| *Streptococcus* | 0.02 | 0.03 | 0.01 | 0.03 | 0.02 | 0.02 | 0.02 | 0.01 | 0.03 | 0.01 | 0.01 | 0.02 | 0.01 | 0.01 |
| Veillonellaceae (unclassified) | 0.35 | 0.41 | 0.33 | 0.27 | 0.32 | 0.37 | 0.37 | 0.33 | 0.57 | 0.43 | 0.58 | 0.54 | 0.33 | 0.52 |
| **Proteobacteria** |  |  |  |  |  |  |  |  |  |  |  |  |  |  |
| Alphaproteobacteria (unclassified) | 0.12 | 0.24 | 0.21 | 0.21 | 0.14 | 0.15 | 0.16 | 0.12 | 0.18 | 0.18 | 0.19 | 0.16 | 0.17 | 0.22 |
| GMD14H09 (unclassified) | 0.32 | 0.26 | 0.23 | 0.22 | 0.3 | 0.35 | 0.33 | 0.3 | 0.42 | 0.29 | 0.24 | 0.37 | 0.31 | 0.25 |
| Rickettsiales (unclassified) | 0.08 | 0.12 | 0.08 | 0.09 | 0.11 | 0.12 | 0.11 | 0.09 | 0.14 | 0.13 | 0.14 | 0.14 | 0.11 | 0.1 |
| *Sutterella* | 0.01 | 0.03 | 0.03 | 0.03 | 0.02 | 0.02 | 0.01 | 0.03 | 0.03 | 0.03 | 0.04 | 0.03 | 0.04 | 0.04 |
| **Spirochaetes** |  |  |  |  |  |  |  |  |  |  |  |  |  |  |
| *Sphaerochaeta* | 0.07 | 0.11 | 0.12 | 0.14 | 0.08 | 0.07 | 0.08 | 0.07 | 0.1 | 0.11 | 0.08 | 0.08 | 0.09 | 0.1 |
| *Treponema* | 4.3 | 2.26 | 2.34 | 2.57 | 2.09 | 2.02 | 2.17 | 4.31 | 2.6 | 2.49 | 2.07 | 2.78 | 2.85 | 2.64 |
| **Synergistetes** |  |  |  |  |  |  |  |  |  |  |  |  |  |  |
| Synergistales (unclassified) | 0.12 | 0.21 | 0.14 | 0.18 | 0.18 | 0.15 | 0.14 | 0.11 | 0.14 | 0.13 | 0.13 | 0.15 | 0.19 | 0.15 |
| **Tenericutes** |  |  |  |  |  |  |  |  |  |  |  |  |  |  |
| Anaeroplasmataceae (unclassified) | 0.07 | 0.07 | 0.08 | 0.05 | 0.05 | 0.03 | 0.06 | 0.05 | 0.1 | 0.09 | 0.1 | 0.07 | 0.06 | 0.06 |
| Anaeroplasma | 0.1 | 0.15 | 0.17 | 0.11 | 0.11 | 0.13 | 0.07 | 0.13 | 0.19 | 0.15 | 0.14 | 0.15 | 0.13 | 0.16 |
| Mollicutes (unclassified) | 0.02 | 0.04 | 0.05 | 0.05 | 0.03 | 0.04 | 0.03 | 0.04 | 0.05 | 0.05 | 0.04 | 0.04 | 0.04 | 0.05 |
| Mycoplasmataceae (unclassified) | 0.02 | 0.02 | 0.01 | 0 | 0.01 | 0.02 | 0.01 | 0.01 | 0.01 | 0.01 | 0.01 | 0.02 | 0.01 | 0.02 |
| RF39 (unclassified) | 0.04 | 0.07 | 0.07 | 0.08 | 0.05 | 0.06 | 0.04 | 0.06 | 0.06 | 0.06 | 0.07 | 0.05 | 0.06 | 0.05 |
| **Verrucomicrobia** |  |  |  |  |  |  |  |  |  |  |  |  |  |  |
| RFP12 (unclassified) | 0.02 | 0.06 | 0.07 | 0.04 | 0.04 | 0.06 | 0.05 | 0.04 | 0.06 | 0.04 | 0.06 | 0.07 | 0.07 | 0.07 |

Genera with >75% presence across all samples
