# Supplementary material for: Assessment of fecal bacterial viability and diversity in fresh and frozen fecal microbiota transplant (FMT) product in horses
Source: BMC Vet Res. 2024 Jul 10;20:306. doi: 10.1186/s12917-024-04166-w (PMC11234551; doi:10.1186/s12917-024-04166-w)
Supplement: Supplementary file 11 — Additional Table 5: Difference of relative abundance for genera common to both DNA-based and cDNA-based analysis at day 0 (fresh sample) [file 12917_2024_4166_MOESM11_ESM.docx]

| **Phylum**  *Genus* | **P-value** |
| --- | --- |
| **Bacteria (unclassified)** | <0.001 |
| **Actinobacteria** |  |
| *Adlercreutzia* | <0.001 |
| Coriobacteriaceae 1 (unclassified) | <0.001 |
| Coriobacteriaceae 1 (unclassified) | <0.001 |
| **Armatimonadetes** |  |
| RB046 (unclassified) | <0.001 |
| **Bacteroidetes** |  |
| Bacteroidales 1 (unclassified) | <0.001 |
| Bacteroidales 2 (unclassified) | <0.001 |
| *Bacteroides* | <0.001 |
| *BF311* | <0.001 |
| *CF231* | <0.001 |
| Marinilabiaceae (unclassified) | <0.001 |
| *Paludibacter* | <0.001 |
| Paraprevotellaceae 1 (unclassified) | <0.001 |
| Paraprevotellaceae 2 (unclassified) | <0.001 |
| *Prevotella 1* | <0.001 |
| *Prevotella 2* | <0.001 |
| RF16 (unclassified) | <0.001 |
| S24-7 (unclassified) | <0.001 |
| *YRC22* | <0.001 |
| **Cyanobacteria** |  |
| YS2 (unclassified) | <0.001 |
| **Fibrobacteres** |  |
| *Fibrobacter* | <0.001 |
| **Firmicutes** |  |
| *Anaerovibrio* | <0.01 |
| *Bulleidia* | <0.001 |
| Christensenellaceae (unclassified) | <0.001 |
| Clostridiales 1 (unclassified) | <0.001 |
| Clostridiales 2 (unclassified) | <0.01 |
| *Clostridium* | <0.001 |
| *Coprococcus* | <0.001 |
| *Dorea* | <0.001 |
| *Epulopiscium* | <0.001 |
| Erysipelotrichaceae 1 (unclassified) | <0.001 |
| Erysipelotrichaceae 2 (unclassified) | <0.001 |
| *Eubacterium* | <0.001 |
| Firmicutes (unclassified) | <0.001 |
| Lachnospiraceae 1 (unclassified) | <0.001 |
| Lachnospiraceae 2 (unclassified) | <0.001 |
| *Lactobacillus* | <0.001 |
| Mogibacteriaceae (unclassified) | <0.001 |
| *Oscillospira* | <0.001 |
| *p-75-a5* | <0.001 |
| *Phascolarctobacterium* | <0.001 |
| *Pseudoramibacter Eubacterium* | <0.001 |
| *RFN20* | <0.001 |
| *Roseburia* | <0.001 |
| Ruminococcaceae 1 (unclassified) | <0.001 |
| Ruminococcaceae 2 (unclassified) | <0.001 |
| *Ruminococcus* | <0.001 |
| *Streptococcus* | 0.01 |
| Veillonellaceae (unclassified) | <0.001 |
| **Proteobacteria** |  |
| Alphaproteobacteria (unclassified) | <0.001 |
| GMD14H09 (unclassified) | 0.58 |
| Rickettsiales (unclassified) | <0.001 |
| *Sutterella* | <0.001 |
| **Spirochaetes** |  |
| *Sphaerochaeta* | <0.001 |
| *Treponema* | <0.001 |
| **Synergistetes** |  |
| Synergistales (unclassified) | <0.001 |
| **Tenericutes** |  |
| *Anaeroplasma* | <0.001 |
| Anaeroplasmataceae (unclassified) | <0.001 |
| Mollicutes (unclassified) | <0.001 |
| Mycoplasmataceae (unclassified) | <0.001 |
| RF39 (unclassified) | <0.001 |
| **Verrucomicrobia** |  |
| RFP12 (unclassified) | <0.001 |

P-value <0.05 considered significant
