# Supplementary material for: Assessment of fecal bacterial viability and diversity in fresh and frozen fecal microbiota transplant (FMT) product in horses
Source: BMC Vet Res. 2024 Jul 10;20:306. doi: 10.1186/s12917-024-04166-w (PMC11234551; doi:10.1186/s12917-024-04166-w)
Supplement: Supplementary file 12 — Additional Table 6: Effect of storage variables and their interactions on mean relative abundance of common genera between DNA-based and cDNA-based analysis within each frozen storage temperature [file 12917_2024_4166_MOESM12_ESM.docx]

|  | **DNA** | | | | | | **cDNA** | | | | | |
| --- | --- | --- | --- | --- | --- | --- | --- | --- | --- | --- | --- | --- |
|  | **-20** | | | **-80** | | | **-20** | | | **-80** | | |
| **Genus** | **Buffer** | **Day** | **Buffer***  **Day** | **Buffer** | **Day** | **Buffer***  **Day** | **Buffer** | **Day** | **Buffer***  **Day** | **Buffer** | **Day** | **Buffer**  *** Day** |
| Actinobacteria |  |  |  |  |  |  |  |  |  |  |  |  |
| Adlercreutzia | 0.678 | <0.001 | <0.001 | 0.382 | <0.001 | <0.001 | 0.032 | <0.001 | <0.001 | 0.001 | <0.001 | <0.001 |
| Coriobacteriaceae Unclassified 1 | 0.247 | 0.013 | 0.944 | 0.652 | <0.001 | 0.174 | 0.162 | 0.368 | 0.873 | 0.584 | 0.101 | 0.995 |
| Coriobacteriaceae Unclassified 2 | 0.760 | 0.099 | 0.115 | 0.990 | 0.001 | 0.010 | 0.162 | <0.001 | <0.001 | 0.503 | <0.001 | 0.206 |
| Armatimonadetes |  |  |  |  |  |  |  |  |  |  |  |  |
| RB046 Unclassified | 0.981 | 0.051 | 0.274 | 0.633 | 0.707 | 0.936 | 0.941 | <0.001 | 0.203 | 0.336 | 0.149 | 0.576 |
| Bacteria |  |  |  |  |  |  |  |  |  |  |  |  |
| Unclassified Bacteria 1 | 0.086 | <0.001 | <0.001 | 0.007 | <0.001 | <0.001 | 0.577 | <0.001 | <0.001 | 0.018 | <0.001 | <0.001 |
| Bacteroidetes |  |  |  |  |  |  |  |  |  |  |  |  |
| [Paraprevotellaceae] Unclassified 1 | 0.124 | 0.064 | 0.409 | 0.131 | 0.693 | 0.694 | 0.347 | 0.598 | 0.144 | 0.128 | 0.253 | 0.081 |
| [Paraprevotellaceae] Unclassified 2 | 0.423 | 0.001 | 0.013 | 0.418 | 0.727 | 0.725 | 0.081 | 0.027 | 0.003 | 0.351 | 0.052 | 0.103 |
| [Prevotella] | 0.011 | 0.811 | 0.025 | 0.014 | 0.021 | 0.007 | 0.577 | 0.015 | 0.003 | 0.079 | 0.052 | 0.033 |
| Bacteroidales Unclassified 1 | 0.033 | <0.001 | <0.001 | 0.415 | 0.061 | 0.004 | <0.001 | <0.001 | 0.002 | 0.018 | <0.001 | <0.001 |
| Bacteroidales Unclassified 2 | <0.001 | <0.001 | <0.001 | 0.195 | 0.002 | 0.001 | <0.001 | 0.043 | 0.014 | 0.065 | 0.019 | 0.003 |
| Bacteroides | 0.007 | 0.305 | 0.207 | 0.014 | 0.030 | 0.089 | 0.087 | 0.315 | 0.991 | 0.189 | 0.888 | 0.635 |
| BF311 | 0.462 | 0.003 | 0.091 | 0.526 | <0.001 | 0.001 | <0.001 | <0.001 | 0.600 | <0.001 | 0.307 | <0.001 |
| CF231 | 0.861 | 0.380 | 0.306 | 0.693 | 0.001 | 0.012 | 0.727 | 0.016 | <0.001 | 0.007 | 0.067 | <0.001 |
| Marinilabiaceae Unclassified | 0.331 | <0.001 | 0.018 | 0.158 | 0.145 | 0.573 | 0.259 | <0.001 | <0.001 | 0.273 | <0.001 | <0.001 |
| Paludibacter | <0.001 | <0.001 | <0.001 | <0.001 | <0.001 | <0.001 | <0.001 | <0.001 | 0.004 | <0.001 | <0.001 | <0.001 |
| Prevotella | 0.011 | <0.001 | <0.001 | 0.026 | 0.298 | 0.025 | 0.003 | 0.009 | 0.012 | 0.001 | <0.001 | 0.001 |
| RF16 Unclassified | <0.001 | <0.001 | <0.001 | <0.001 | <0.001 | <0.001 | 0.272 | 0.921 | 0.001 | <0.001 | <0.001 | 0.856 |
| S24-7 Unclassified | 0.030 | <0.001 | <0.001 | 0.015 | 0.027 | 0.003 | 0.048 | 0.140 | 0.425 | <0.001 | 0.004 | <0.001 |
| YRC22 | <0.001 | 0.171 | 0.004 | 0.020 | <0.001 | <0.001 | 0.306 | 0.015 | 0.365 | 0.706 | 0.061 | 0.245 |
| Cyanobacteria |  |  |  |  |  |  |  |  |  |  |  |  |
| YS2 Unclassified | 0.291 | 0.023 | 0.522 | 0.045 | <0.001 | <0.001 | <0.001 | <0.001 | <0.001 | <0.001 | <0.001 | 0.467 |
| Fibrobacteres |  |  |  |  |  |  |  |  |  |  |  |  |
| Fibrobacter | <0.001 | <0.001 | <0.001 | <0.001 | <0.001 | <0.001 | <0.001 | <0.001 | <0.001 | 0.003 | <0.001 | <0.001 |
| Firmicutes |  |  |  |  |  |  |  |  |  |  |  |  |
| [Eubacterium] | 0.033 | <0.001 | <0.001 | 0.020 | <0.001 | <0.001 | <0.001 | <0.001 | <0.001 | 0.843 | <0.001 | 0.701 |
| [Mogibacteriaceae] Unclassified 2 | 0.001 | <0.001 | <0.001 | 0.007 | <0.001 | <0.001 | <0.001 | <0.001 | <0.001 | <0.001 | 0.001 | 0.010 |
| Anaerovibrio | 0.423 | 0.004 | 0.189 | 0.571 | 0.466 | 0.448 | 0.016 | 0.017 | 0.010 | <0.001 | 0.141 | 0.117 |
| Bulleidia | 0.058 | 0.051 | 0.571 | 0.007 | <0.001 | <0.001 | 0.709 | 0.368 | 0.769 | 0.532 | 0.029 | 0.002 |
| Christensenellaceae Unclassified 2 | 0.794 | 0.005 | 0.085 | 0.498 | 0.009 | 0.037 | 0.090 | 0.272 | 0.026 | 0.843 | 0.020 | 0.995 |
| Clostridiales Unclassified 1 | <0.001 | <0.001 | <0.001 | <0.001 | <0.001 | 0.049 | <0.001 | <0.001 | <0.001 | 0.212 | <0.001 | <0.001 |
| Clostridiales Unclassified 2 | 0.036 | <0.001 | <0.001 | 0.258 | <0.001 | <0.001 | 0.575 | <0.001 | 0.389 | 0.206 | 0.319 | <0.001 |
| Clostridium 1 | 0.001 | 0.081 | 0.762 | 0.039 | <0.001 | 0.003 | 0.575 | <0.001 | <0.001 | 0.299 | <0.001 | <0.001 |
| Coprococcus | <0.001 | <0.001 | <0.001 | <0.001 | 0.819 | 0.171 | 0.709 | 0.046 | <0.001 | 0.703 | 0.008 | <0.001 |
| Dorea | 0.981 | 0.009 | 0.668 | 0.382 | 0.744 | 0.339 | 0.090 | 0.007 | 0.174 | 0.351 | 0.528 | 0.701 |
| Epulopiscium | 0.050 | 0.001 | 0.050 | 0.006 | 0.191 | 0.171 | 0.004 | <0.001 | <0.001 | <0.001 | <0.001 | <0.001 |
| Erysipelotrichaceae Unclassified 1 | 0.074 | 0.122 | 0.158 | 0.128 | 0.191 | 0.448 | 0.709 | 0.239 | 0.744 | 0.839 | 0.999 | 0.995 |
| Erysipelotrichaceae Unclassified 2 | 0.015 | 0.423 | 0.977 | 0.016 | 0.285 | 0.604 | 0.631 | 0.953 | 0.884 | 0.703 | 0.688 | 0.016 |
| Lachnospiraceae Unclassified 1 | 0.423 | <0.001 | <0.001 | 0.001 | <0.001 | 0.003 | <0.001 | <0.001 | <0.001 | <0.001 | <0.001 | <0.001 |
| Lachnospiraceae Unclassified 2 | 0.073 | <0.001 | <0.001 | 0.028 | <0.001 | <0.001 | <0.001 | <0.001 | 0.741 | <0.001 | <0.001 | <0.001 |
| Lactobacillus | <0.001 | <0.001 | 0.001 | <0.001 | <0.001 | <0.001 | 0.709 | 0.313 | 0.541 | 0.059 | 0.997 | 0.079 |
| Oscillospira | 0.794 | 0.949 | 0.074 | 0.791 | 0.337 | 0.019 | 0.866 | <0.001 | <0.001 | 0.036 | 0.003 | 0.995 |
| p-75-a5 | 0.750 | 0.003 | 0.035 | 0.439 | 0.850 | 0.694 | 0.034 | <0.001 | 0.014 | 0.843 | <0.001 | 0.273 |
| Phascolarctobacterium | 0.794 | <0.001 | <0.001 | 0.011 | <0.001 | <0.001 | <0.001 | <0.001 | 0.017 | 0.703 | 0.688 | 0.995 |
| Pseudoramibacter_Eubacterium | 0.011 | 0.982 | 0.159 | 0.039 | 0.145 | 0.958 | <0.001 | <0.001 | <0.001 | 0.002 | <0.001 | <0.001 |
| RFN20 | <0.001 | <0.001 | <0.001 | <0.001 | <0.001 | <0.001 | 0.306 | 0.001 | 0.265 | 0.832 | <0.001 | <0.001 |
| Roseburia | 0.150 | 0.982 | 0.091 | 0.192 | <0.001 | <0.001 | <0.001 | 0.726 | 0.879 | 0.003 | <0.001 | <0.001 |
| Ruminococcaceae Unclassified 1 | 0.046 | 0.007 | 0.028 | 0.007 | 0.003 | 0.008 | 0.860 | <0.001 | 0.039 | <0.001 | <0.001 | <0.001 |
| Ruminococcaceae Unclassified 2 | <0.001 | <0.001 | <0.001 | <0.001 | <0.001 | <0.001 | 0.032 | <0.001 | <0.001 | <0.001 | <0.001 | <0.001 |
| Ruminococcus | <0.001 | 0.647 | 0.001 | <0.001 | 0.172 | 0.879 | 0.002 | <0.001 | <0.001 | 0.128 | 0.954 | <0.001 |
| Streptococcus | 0.794 | 0.055 | 0.465 | 0.981 | 0.214 | 0.812 | 0.511 | 0.213 | 0.849 | 0.561 | 0.815 | 0.530 |
| Unclassified | 0.030 | 0.051 | 0.014 | 0.195 | 0.218 | 0.464 | 0.003 | 0.095 | 0.006 | <0.001 | <0.001 | <0.001 |
| Veillonellaceae Unclassified 2 | 0.047 | <0.001 | <0.001 | 0.498 | 0.869 | 0.807 | <0.001 | <0.001 | <0.001 | 0.209 | 0.498 | 0.033 |
| Proteobacteria |  |  |  |  |  |  |  |  |  |  |  |  |
| Alpha Unclassified 2 | <0.001 | <0.001 | <0.001 | <0.001 | <0.001 | <0.001 | 0.011 | 0.005 | 0.563 | 0.206 | 0.311 | 0.002 |
| GMD14H09 Unclassified | 0.017 | 0.200 | 0.977 | 0.005 | 0.727 | 0.215 | 0.866 | <0.001 | 0.001 | 0.412 | 0.097 | 0.103 |
| Rickettsiales Unclassified | 0.045 | 0.064 | 0.078 | 0.068 | 0.025 | 0.080 | 0.709 | 0.826 | 0.026 | 0.678 | 0.075 | 0.480 |
| Sutterella | 0.021 | 0.985 | 0.529 | 0.001 | 0.191 | 0.004 | 0.666 | 0.393 | 0.884 | 0.890 | 0.074 | 0.018 |
| Spirochaetes |  |  |  |  |  |  |  |  |  |  |  |  |
| Sphaerochaeta | 0.678 | 0.019 | 0.388 | 0.353 | <0.001 | <0.001 | 0.577 | <0.001 | 0.144 | 0.637 | 0.451 | 0.493 |
| Treponema | <0.001 | 0.005 | 0.139 | <0.001 | <0.001 | 0.014 | <0.001 | <0.001 | <0.001 | 0.336 | <0.001 | <0.001 |
| Synergistetes |  |  |  |  |  |  |  |  |  |  |  |  |
| Synergistales Unclassified | 0.001 | 0.687 | 0.529 | <0.001 | 0.065 | 0.032 | 0.162 | 0.002 | 0.011 | <0.001 | 0.084 | 0.001 |
| Tenericutes |  |  |  |  |  |  |  |  |  |  |  |  |
| Anaeroplasma | 0.247 | 0.001 | 0.013 | 0.791 | <0.001 | <0.001 | 0.001 | 0.005 | 0.060 | 0.371 | 0.009 | 0.048 |
| Anaeroplasmataceae Unclassified 2 | 0.394 | 0.876 | 0.857 | 0.981 | <0.001 | <0.001 | 0.314 | 0.548 | 0.004 | 0.447 | 0.029 | 0.050 |
| Mollicutes Unclassified 2 | 0.131 | 0.914 | 0.938 | 0.505 | <0.001 | 0.001 | <0.001 | <0.001 | 0.003 | 0.019 | 0.020 | 0.245 |
| Mycoplasmataceae Unclassified | <0.001 | <0.001 | <0.001 | <0.001 | <0.001 | <0.001 | 0.866 | 0.017 | 0.541 | 0.706 | 0.873 | 0.995 |
| RF39 Unclassified | 0.838 | 0.200 | 0.725 | 0.697 | <0.001 | 0.011 | 0.002 | <0.001 | 0.001 | 0.528 | 0.873 | 0.783 |
| Verrucomicrobia |  |  |  |  |  |  |  |  |  |  |  |  |
| RFP12 Unclassified | 0.001 | <0.001 | 0.762 | 0.007 | <0.001 | 0.088 | 0.076 | 0.010 | 0.172 | <0.001 | <0.001 | 0.032 |
